# Evolution and spillover dynamics of yellow fever at the forest–urban interface in Brazil

**DOI:** 10.1038/s41564-026-02302-w

**Published:** 2026-03-11

**Authors:** Juliana Telles-de-Deus, Ingra M. Claro, Mayara Bertanhe, Charles Whittaker, Márcio Port-Carvalho, Esmenia C. Rocha, Thaís M. Coletti, Camila A. M. da Silva, Ian Nunes Valença, Tamara N. Lima-Camara, Márcia Bicudo de Paula, Mariana S. Cunha, Jaqueline G. de Jesus, Pâmela dos Santos Andrade, Victoria Cox, Natalia C. C. F. de Azevedo, Juliana M. Guerra, Juliana L. Summa, Ana Paula P. Teixeira, Eduardo S. Bergo, Mariza Pereira, Filipe R. R. Moreira, Alvina Clara Felix, Anderson V. de Paula, Raissa H. de Araujo Eliodoro, Marissa da Silva Lima, Franciane M. de Oliveira, Valquíria R. de Souza, Lucas A. M. Franco, Marcelo S. Nardi, Thais C. Sanches, Eric T. B. C. da Silva, Amanda A. C. Coimbra, Paulo R. dos Santos, Katherine Lima de Gouveia, Francisco E. S. P. Vilela, Sarah C. Hill, Dilmar A. G. Oliveira, Hélia M. Piedade, Thaís Guimarães-Luiz, Camila M. G. Abreu, Guilherme Casoni da Rocha, Leandro Abade, William M. de Souza, Ben Lambert, Renato Pereira de Souza, Adriano Pinter, Ester C. Sabino, Luis Filipe Mucci, Nuno R. Faria

**Affiliations:** 1 Secretaria de Estado da Saúde de São Paulo, Instituto Pasteur, São Paulo, Brazil; 2 Departamento de Moléstias Infecciosas e Parasitárias, Faculdade de Medicina da Universidade de São Paulo, São Paulo, Brazil; 3 Instituto de Medicina Tropical, Faculdade de Medicina da Universidade de São Paulo, São Paulo, Brazil; 4 MRC Centre for Global Infectious Disease Analysis, School of Public Health, Imperial College London, London, UK; 5 Department of Microbiology, Immunology, and Molecular Genetics, College of Medicine, University of Kentucky, Lexington, KY USA; 6 Division of Infectious Diseases & Vaccinology, School of Public Health, University of California Berkeley, Berkeley, CA USA; 7 Conservation Biodiversity Nucleus, Environmental Research Institute, Secretariat for the Environment, Infrastructure and Logistics of São Paulo, São Paulo, Brazil; 8 Post-Graduation Program in Biodiversity of Conservations Units, National School of Tropical Botanic, Rio de Janeiro Botanical Garden, Rio de Janeiro, Brazil; 9 Departamento de Epidemiologia, Faculdade de Saúde Pública, Universidade de São Paulo, São Paulo, Brazil; 10 Centro de Virologia, Núcleo de Doenças de Transmissão Vetorial, Instituto Adolfo Lutz, São Paulo, Brazil; 11 Núcleo de Anatomia Patológica, Instituto Adolfo Lutz, São Paulo, Brazil; 12 Divisão da Fauna Silvestre, Secretaria do Verde e Meio Ambiente da Prefeitura de São Paulo, São Paulo, Brazil; 13 São Paulo Municipal Department of Health, São Paulo, Brazil; 14 Departamento de Genética, Instituto de Biologia, Universidade Federal do Rio de Janeiro, Rio de Janeiro, Brazil; 15 Parque Estadual Alberto Löfgren, Secretaria do Meio Ambiente do Governo do Estado de São Paulo, São Paulo, São Paulo, Brazil; 16 Department of Pathobiology and Population Sciences, Royal Veterinary College, Hatfield, UK; 17 Departamento de Gestão da Fauna Silvestre, Coordenadoria de Fauna Silvestre, Secretaria de Meio Ambiente, Infraestrutura e Logística do Estado de São Paulo, São Paulo, Brazil; 18 St Mary’s University, Twickenham London, Twickenham, UK; 19 Department of Statistics, University of Oxford, Oxford, UK; 20 Pandemic Sciences Institute, University of Oxford, Oxford, UK; 21 Centro de Laboratório Regional XII, Núcleo de Ciências Biológicas, Instituto Adolfo Lutz, Taubaté, São Paulo Brazil; 22 Departamento de Medicina Veterinária Preventiva e Saúde Animal, Faculdade de Medicina Veterinária e Zootecnia, Universidade de São Paulo, São Paulo, Brazil

**Keywords:** Molecular evolution, Population dynamics, Viral infection

## Abstract

Yellow fever virus (YFV) continues to threaten human and wildlife populations in the Americas, yet its transmission at the forest–urban interface remains unclear. Here we integrate ground- and canopy-level mosquito surveillance, systematic monitoring of non-human primate carcasses and viral metagenomics to describe the dynamics of a sylvatic YFV outbreak in a 186-hectare Atlantic Forest fragment embedded within metropolitan São Paulo, Brazil, between 2017 and 2018. Our analyses reveal that transmission was primarily driven by a single genetic cluster introduced during a period of high abundance of the main vector, *Haemagogus leucocelaenus* mosquitoes. A near-complete hepatitis A virus genome was detected in a YFV-infected howler monkey, suggesting potential co-infections at the human–wildlife interface. Phylogenetic and epidemiological modelling estimated a basic reproduction number, *R*_0_, for sylvatic yellow fever of 8.2 (95% CI 5.1–12.2), substantially higher than previous estimates for urban outbreaks. Our findings demonstrate that multisource surveillance could provide actionable early warnings in regions at risk for zoonotic spillover.

## Abstract (web summary)

Integration of mosquito and non-human primate surveillance and viral metagenomics reveals yellow fever virus outbreak dynamics at an urban–forest interface in Brazil.

## Main

Yellow fever is a mosquito-borne viral haemorrhagic disease with an estimated 47% case fatality rate among non-vaccinated humans^1^. It is caused by the yellow fever virus (YFV, *Orthoflavivirus flavi*), a single-stranded 10.86 kb RNA flavivirus related to dengue, Zika and West Nile viruses. In Africa, YFV circulates in alternating enzootic cycles maintained among non-human primates (NHPs) and small mammals via *Aedes africanus*, and during urban cycles the virus is transmitted by *Aedes aegypti*^2^. In the Americas, urban transmission has not been reported since 1942^3^, and the earliest evidence of a sylvatic YFV cycle dates to 1932^4^. During sylvatic cycles, arboreal *Haemagogus* and *Sabethes* mosquitoes transmit YFV among susceptible NHPs that act as amplifying hosts. These mosquitoes occasionally feed at ground level, increasing the risk of spillover to humans at forest–urban interfaces^5^.

In the Americas, YFV persists in an enzootic cycle in the Amazon and periodically spreads southeastward to densely populated states^6,7^. In 2015, it reached southeastern Brazil, causing 772 human deaths and over 15,000 NHP deaths between 2016 and 2018^8^. Entomological surveys identified *Haemagogus leucocelaenus* and *Haemagogus janthinomys* mosquitoes as primary vectors^5^. Among Brazilian NHPs, howler monkeys (*Alouatta guariba*, family Atelidae) are particularly susceptible to YFV^9,10^. Because NHP cases often precede human cases by several days^11^, tracking NHP cases is critical for guiding ring vaccination programmes, particularly in periods of vaccine shortage.

YFV spillover risk is highest where human activity overlaps with suitable ecologies for vectors and NHPs^12^. Recurrent sylvatic outbreaks, detection of YFV in *Aedes albopictus* and laboratory competence of urban vectors have long raised concerns about renewed urban transmission^13^. Howler monkey populations, once widely distributed across eight states, continue to decline from repeated epizootics, habitat loss and hunting^14^. Tracking YFV in arboreal mosquitoes and NHPs is hampered by logistical constraints, absence of rapid diagnostic tests and challenges in sampling at tree-canopy levels^15^. At broad scales, warmer and wetter conditions and fragmented forest–cropland landscapes rich in susceptible NHPs may accelerate YFV spread and spillover^16^. Yet, at higher resolutions, YFV transmission dynamics remains poorly understood, particularly in high-risk areas such as forest fragments near large urban centres. These gaps reflect challenges in entomological and epidemiological data collection, and the absence of frameworks for modelling YFV zoonotic transmission^17^.

We integrated field mosquito and NHP sampling with viral metagenomics, using a pathogen-agnostic sequencing approach enabling recovery of complete viral genomes^18^, and combined these data with phylogenetic and transmission models to characterize YFV epizootic spread and dynamics during the first YFV outbreak in metropolitan São Paulo.

## Results

### Early outbreak investigations

**Fig. 1:**
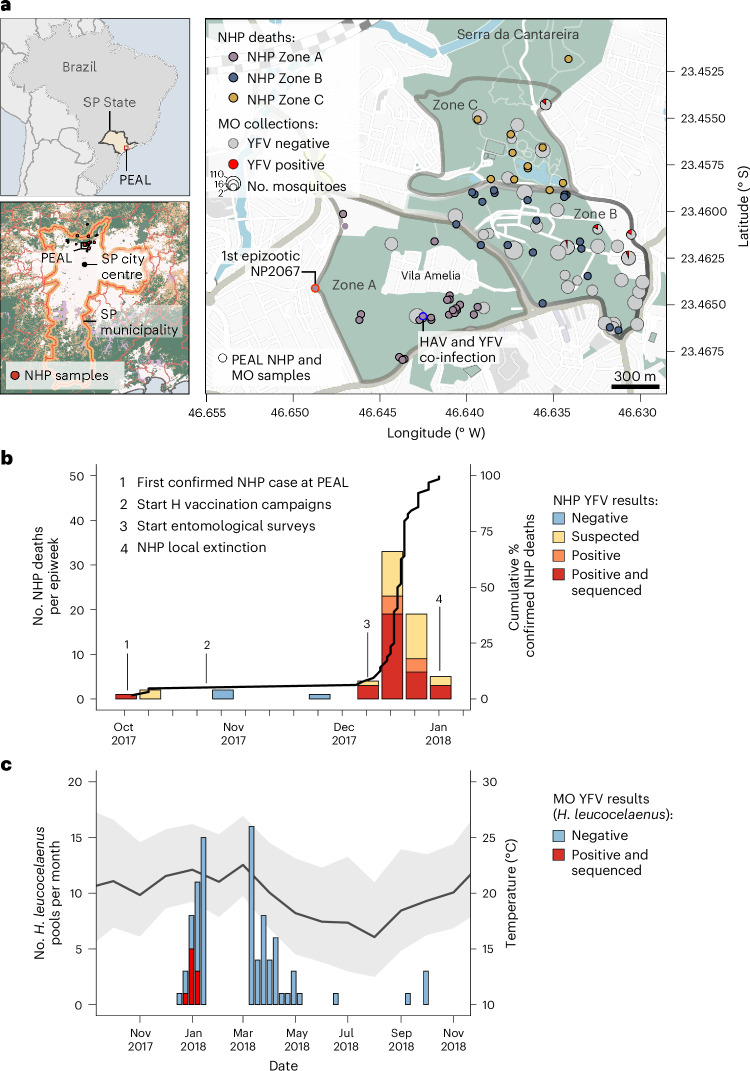
Spatial and temporal context of the YFV epizootic in PEAL’s State Park (São Paulo, Brazil). **a**, Location of PEAL within Brazil (top left) and within São Paulo metropolitan area (bottom left; surrounding biomes from MapBiomas^120^; forested areas, green; farming, orange; freshwater, purple; non-forested natural formations, light brown). Right: spatial distribution of non-human primate (NHP) deaths and mosquito sampling across PEAL management zones (A–C). Red-outlined circle marks the first PEAL epizootic case (NP2067); blue-outlined circle marks the NHP co-infected with YFV and HAV (NP2754). Pie charts indicate mosquito pool sites, with the red sector indicating YFV-positive pools and circle area proportional to the number of sampled pools (smallest circle *n* = 2). NHP deaths are scaled by the number of carcasses (smallest *n* = 1). Note that **a** shows the collection of all mosquito taxa, not only *H. leucocelaenus*. Zones A, B and C correspond to different management areas within PEAL State Park, as defined by the official map from the Government of São Paulo. **b**, Epidemic curve of free-ranging *A. guariba* deaths at PEAL by epidemiological week from first epizootic case (NP2067). Bars are stacked by outcome (negative, suspected, positive, positive + sequenced); black line shows the cumulative percentage of deaths among the free-ranging *A. guariba* population at PEAL. Numbers indicate key events. **c**, Timeseries of non-blood-fed *H. leucocelaenus* pools collected at PEAL, coloured by RT–PCR YFV result (negative, positive + sequenced). The dark grey line and light grey shading shows mean temperature (°C) and minimum/maximum ranges from ERA5-Land^121^, illustrating seasonal conditions during mosquito surveillance. H, human; MO, mosquito; SP, São Paulo.

Between October 2017 and January 2018, an unprecedent yellow fever outbreak occurred at Parque Estadual Alberto Lofgren (PEAL) (Fig. 1a). The first detected epizootic, a juvenile *Alouatta guariba* carcass (NP2067) found on 9 October 2017, marked the first case in the Greater Metropolitan Region of São Paulo (home to >23 million inhabitants) and led to PEAL’s closure and initiation of human vaccination campaigns in adjacent neighbourhoods on 20 October 2017 (Fig. 1b). Entomological surveys near NP2067 on 21 October 2017 detected 11 mosquito species, including *A. albopictus*, but none were YFV positive, and *Haemagogus* and *Sabethes* mosquitoes were absent (Fig. 1a).

Before the outbreak, *A. guariba* density at PEAL was 39.08 individuals km^−2^, the highest reported for this species in the Atlantic Forest^19–22^. By 5 January 2018, the entire population of *A. guariba* became locally extinct (Fig. 1b). Retrospective testing found YFV RNA in 39 of 67 carcasses (58.2%), with 3 YFV RNA-negative and 25 unsampled due to advanced decomposition (Fig. 1c). The case fatality ratio (CFR) at PEAL was 0.96 (95% Bayesian confidence intervals [BCI]: 0.91–1.00) including suspected cases (*n* = 64), and 0.58 (95% BCI: 0.43–0.73) based on confirmed cases (*n* = 39).

### *H. leucocelaenus* as the primary YFV vector at PEAL

**Extended Data Fig. 1:**
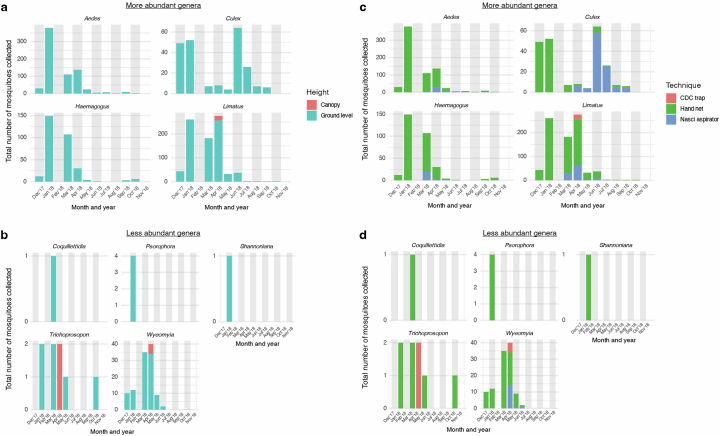
Most and least abundant mosquito genera collected at PEAL stratified by collection technique (a, b) and collection height (c, d). Total number of mosquitoes tested (number of mosquitoes multiplied by pool size) per epidemiological week. The most abundant genera were *Limatus* (37.6%, n = 838), *Aedes* (31.6%, n = 705) – including *Ae. scapularis* (13.3%, n = 297), *Ae. albopictus* (13.1%, n = 292), and *Ae. aegypti* (1.0%, n = 23); *Haemagogus* (14.0%, n = 312), and *Culex* (10.0%, n = 223), while *Sabethes* was less common (1.4%, n = 31). Among 818 pools, 98.7% (n = 807; 2,022 specimens) were collected at ground level and 1.3% (n = 11; 29 specimens) at the canopy level. Raw data, including height of entomological collections, dates and coordinates of each location, are provided in Supplementary Table 1.

**Extended Data Fig. 2:**
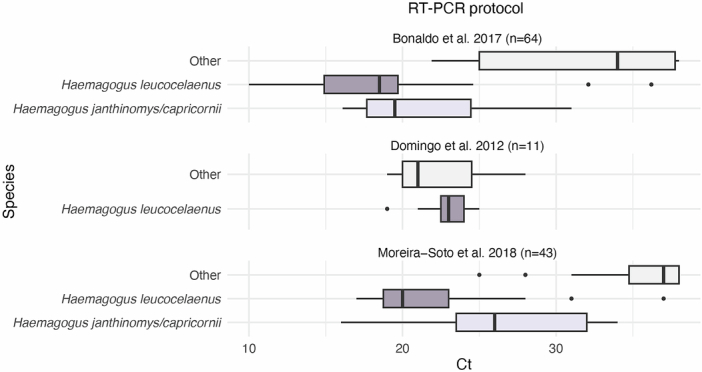
YFV Ct values across vector species. Ct values from YFV-positive mosquito pools by species and RT-qPCR protocols^1^°^5–1^°^7^. Within-protocol comparisons indicate significantly lower Ct values for *Hg. leucocelaenus* (n = 64) compared to *Hg. janthinomys/capricornii* (n = 25) and other species (n = 29). N indicates number of observations. Pools from other species with available YFV cycle threshold values include *Ae. scapularis* (n = 7), *Ae. taeniorhynchus* (n = 1), *Ae. albopictus* (n = 3), *Ae. serratus* (n = 4), *Psorophora ferox* (n = 5), *Sabethes albiprivus* (n = 3), *Sabethes chloropterus* (n = 3), *Sabethes identicus* (n = 1), and *Sabethes purpureus* (n = 2). Raw data, including mosquito species, date of collection, mean pool Ct, study reference, and protocol reference are provided in Supplementary Table 2. Boxplots show the median (centre horizontal line), the 25th–75th percentiles (box), and whiskers extending to 1.5× the interquartile range, with points beyond this plotted as outliers.

**Extended Data Fig. 3:**
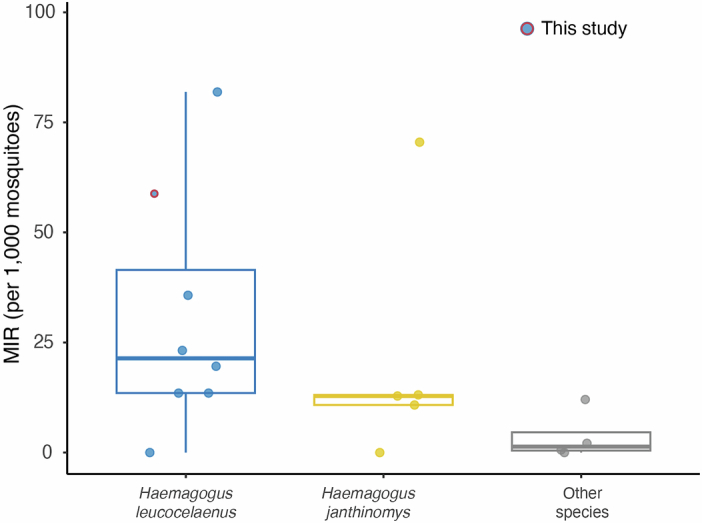
YFV minimum infection rates across vector species. Minimum infection rates (MIR) for YFV vectors in Brazil. Circles show species-specific MIRs obtained from studies in four states (Espírito Santo, Maranhão, Minas Gerais and Rio de Janeiro), obtained from published data^17,38,39^. Only MIR values obtained from testing ≥10 pools per species were included (*Hg. leucocelaenus*: n = 8, *Hg. janthinomys*: n = 5, “Other”: n = 4). Boxplots summarise the distribution for each taxon (median, IQR; whiskers at 1.5× IQR). The MIR for *Hg. leucocelaenus* at PEAL is highlighted with a red-outlined marker. The ‘Other’ category groups additional sylvatic species with ≥10 pools but too few observations for a separate panel (*Aedes scapularis*, *Ae. taeniorhynchus*, *Sabethes soperi* and *Sa. chloropterus)*. MIRs were computed as (number of YFV-positive pools divided by the total number of adult mosquitoes of the same species) × 1,000, following standard methodology^17,39,110^. Data is available in Supplementary Table 3.

We sampled mosquitoes across 39 locations, collecting 2,231 individuals (2,013 females) from 24 species and 10 genera (Supplementary Table 1, Extended Data Fig. 1 and Supplementary Fig. 1). Among 753 pools of non-blood-fed females, YFV RNA was detected exclusively in *H. leucocelaenus*, with 9 of 87 pools collected at ground-level locations (1–5 mosquitoes per pool; median quantitative PCR with reverse transcription (RT–qPCR) cycle threshold (*C*_t_) = 23, range: 19–25) (Supplementary Table 1). To contextualize these findings, we compared *C*_t_ values across 118 pools representing 12 distinct species drawn from publicly available datasets (Supplementary Table 2). *C*_t_ values differed strongly by species, with *H. leucocelaenus* < *H. janthinomys* < other vectors (global Kruskal–Wallis, *P* = 1.1 × 10^−11^; pairwise *q* = 0.033, 3.5 × 10^−11^ and 1.7 × 10^−6^, respectively). A model adjusting for RT–PCR assay and pool size estimated −2.6 *C*_t_ values for *H. leucocelaenus* relative to other vectors (*P* = 0.034; d.f. = 112), consistent with higher viral RNA in *H. leucocelaenus* pools (Extended Data Fig. 2). The minimum infection rate (MIR) for *H. leucocelaenus* was 58.8 per 1,000 (5.88%). Adjusting for pool sizes using a maximum-likelihood approach yielded a similar MIR of 6.3% (95% CI: 3.1–11.3%). The MIR at PEAL was high compared to other Brazilian studies/sites^5^, and we found no evidence that MIR differs across species (two-sided Kruskal–Wallis *P* = 0.058; all pairwise false discovery rate (FDR)-adjusted tests non-significant; Extended Data Fig. 3 and Supplementary Table 3).

### Environmental drivers of vector abundance

To understand the environmental context in which the outbreak unfolded, we examined the climatic drivers of mosquito abundance. Mean temperature was the primary correlate of *H. leucocelaenus* abundance at PEAL (negative-binomial generalized linear model (GLM); pseudo-*R*^2^ = 0.83–0.86; Fig. 1c and Supplementary Tables 4–6). In genus-specific models including temperature and cumulative rainfall, temperature remained strongly associated with *Haemagogus* abundance (rate ratio, RR per 1 °C = 2.82, 95% CI: 1.73–4.73), while rainfall contributed little (RR = 0.96, 95% CI: 0.87–1.05). Because temperature and rainfall were strongly correlated (Supplementary Fig. 2), rainfall effects should be interpreted cautiously. Similar temperature associations were observed for *Aedes* (RR = 2.81, 95% CI: 1.81–4.59; pseudo-*R*^2^ = 0.75) and *Limatus* (RR = 2.71, 95% CI: 1.63–4.69; pseudo-*R*^2^ = 0.58), with no clear added effect of rainfall. For *Culex*, associations were not significant (temperature RR = 0.81, 95% CI 0.42–1.56; rainfall RR = 1.08, 95% CI 0.91–1.27; pseudo-*R*^2^ = 0.11) (Supplementary Table 7).

### Viral metagenomics and co-infection with hepatitis A virus (HAV)

**Extended Data Fig. 4:**
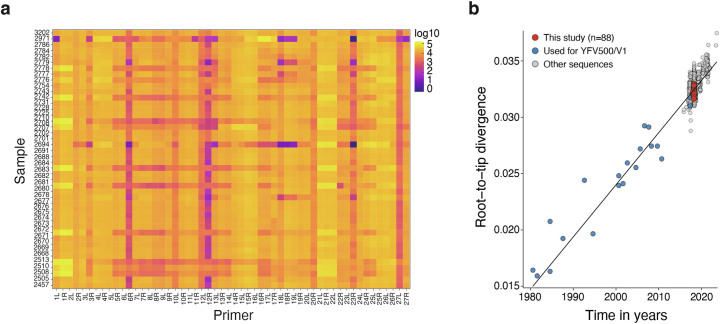
Performance of the YFV500/V1 tiled-amplicon scheme and temporal signal in South American genotype I genomes. (**a**) Primer-binding heatmap across NP samples (n = 42). Each cell shows the number of reads containing a primer (or its reverse complement), normalised by library size (CPM, counts-per-million). Primers are ordered by amplicon number and orientation (L = left; R = right). Lower CPM indicates weaker binding/possible mismatches. (**b**) Root-to-tip genetic divergence against sampling year for South American genotype I genomes (r² = 0.80); n = 1,080 genomes from NCBI GenBank up until April 2023). Red = genomes generated in this study (n = 88); blue = genomes used to design the original YFV500/V1 primer set (up to April 2017); grey = other South American genotype I genomes.

To characterize the performance of our sequencing approaches and to identify any additional viral signals present in the samples, we examined viral metagenomic data and its concordance with tiled‑amplicon sequencing. First, we generated viral metagenomic sequencing data from mosquito pools and NHP tissues^18^ and compared its performance with a tiled-amplicon approach^9^. Metagenomics achieved higher coverage across 56 paired samples (median 99.89% vs 85.99%; mean paired difference 10.75 percentage points, 95% CI 6.70–14.80; two-sided Wilcoxon signed-rank test *P* = 1.18 × 10^−6^) (Supplementary Table 8). Coverage differences widened at lower *C*_t_ values (*n* = 52, *r* = −0.78, *P* = 1.25 × 10^−11^) (Supplementary Fig. 3), partly reflecting primer–template mismatches at 6 sites in the YFV500/V1 scheme (6R, 10L, 12L/12R, 20R, 23R, 27L) and consistent with sequence divergence (Extended Data Fig. 4). In addition, sample pre-treatment (centrifugation, filtration and Turbo DNase) in the viral metagenomics workflow probably further reduced non-viral nucleic acids, further improving viral metagenomic recovery.

Sequencing of 98 YFV RT–PCR-positive samples yielded 88 complete and near-complete YFV sequences with ≥70% coverage. This included all 9 mosquito pools (Group I), 32/36 PEAL NHPs (Group II) and 47/53 background NHPs (Group III) (Supplementary Fig. 4). Overall coverage average, at a sequencing depth of 10× or greater, was high (99.90%, range 72.36–99.98%; Supplementary Tables 8 and 9). Coverage was similarly high in mosquitoes (*n* = 9, median 97.5%, range: 76.2–99.9%) and NHPs (*n* = 89, median 99.90%, range: 0–99.9%). Our metagenomic workflow also generated sufficient mitochondrial reads for cytochrome *c* oxidase subunit I (COI) metabarcoding, enabling species confirmation for 32 *A. guariba* and 4 *H. leucocelaenus* pools.

**Extended Data Fig. 5:**
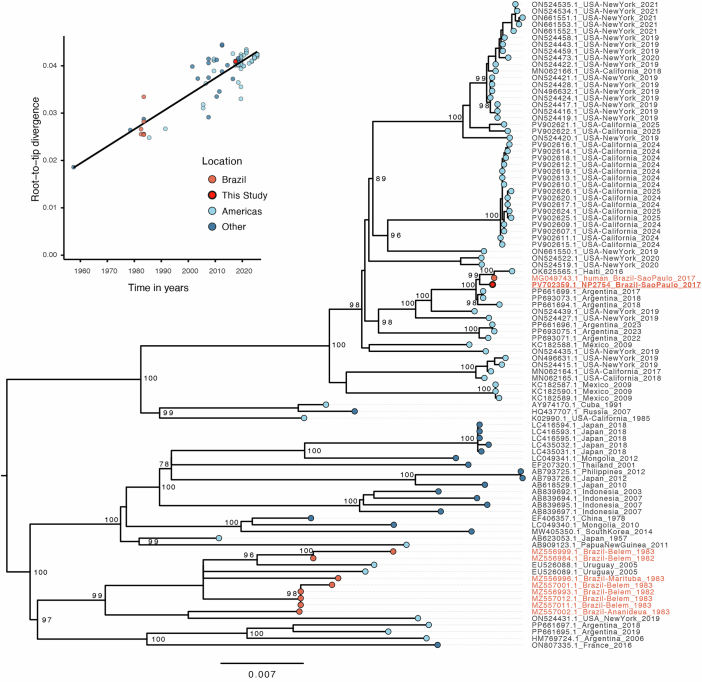
Maximum-likelihood phylogeny of hepatitis A virus (HAV) including the PEAL NP2754 genome. The red tip marks the HAV near complete genome recovered from an *Alouatta guariba* at PEAL (NP2754). Tip colours: dark blue = Brazil (non-PEAL), light blue = other sample collection in the Americas, grey = other global sequences, red = this study. NP2754 nests within the clade that includes a contemporary 2017 São Paulo strain. Numbers on nodes are bootstrap support shown for selected clades. Branch lengths are in substitutions per site units. The tree is mid-point rooted. The map location of NP2754 is shown in Fig. 1a. Inset on the upper left shows root-to-tip regression of genetic divergence against sampling date for the same dataset; points are coloured as above and the line shows the best-fit regression (r^2^ = 0.80, n = 97, sampling range = 68 years).

We also recovered a near-complete HAV genome (73.75% coverage at 10×; lineage HAV I.A_ab) from a YFV-positive female *A. guariba* (NP2754). As *A. guariba* are primarily canopy dwelling, enzootic HAV has not been reported in free-ranging Neotropical primates^23^, and HAV is not handled in our laboratory, this finding most probably reflects environmental exposure to human faecal contamination near the collection site (~100 m from the nearest residence, Fig. 1a). The NP2754 HAV sequence showed 97.1% identity and clustered with strong statistical support with a contemporaneous human strain from São Paulo (Extended Data Fig. 5), consistent with environmental exposure rather than laboratory contamination.

### Association between host and vector viral loads and cycle thresholds

**Fig. 2:**
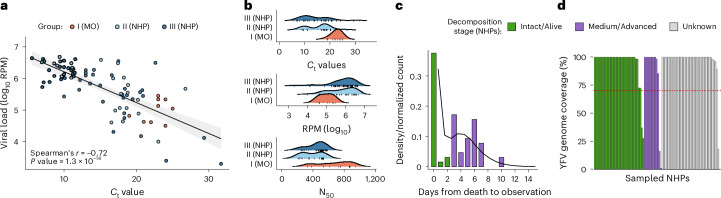
Relationship between RT–PCR *C*_t_ values, viral load, timing of observations and viral detection. **a**, Scatterplot of RT–PCR *C*_t_ values versus YFV mapped RPM for each sample group (Group I: *H. leucocelaenus*, Group II: PEAL NHPs, Group III: background NHPs), illustrating the inverse relationship between *C*_t_ values and viral abundance (Spearman’s *r*, two-sided). Fitted line and shaded band show linear trend and 95% confidence interval. **b**, Density ridge plot displaying the distributions of *C*_t_ values, viral load (RPM) and N_50_ among the different sample groups. **c**, Empirical and estimated distributions of the delay between *A. guariba* death and notification. The black line shows the fitted probability density (gamma distribution). Bars indicate the empirical distribution of days from death to observation, coloured by decomposition stage: green for intact or recently dead animals (0–2 days post death) and purple for medium or advanced decomposition (≥3 days post death) (see Methods). **d**, YFV genome coverage (% of reference) across 89 samples for which viral metagenomics sequencing was attempted (35 from intact or recently dead NHPs; 12 from NHPs found at medium or advanced decomposition; and 43 NHPs for which stage of decomposition was unknown). Samples were grouped by decomposition stage. The horizontal dashed red line marks the 70% whole-genome coverage threshold used for inclusion in phylogenetic analyses. Spearman’s correlations and Wilcoxon tests were two‑sided; exact *P* values are reported in main text.

RT–PCR *C*_t_ values were inversely correlated with YFV reads per million (RPM, log_10_) (*n* = 84, two-sided Spearman’s *r* = −0.72, *P* = 1.3 × 10^−14^; Fig. 2a). NHPs had lower *C*_t_ values than *H. leucocelaenus* pools (*n* = 75, median 14, range: 5.57–31.68 vs *n* = 9, 23, range: 19.00–25.00; Wilcoxon rank-sum *P* = 3.5 × 10^−5^; Fig. 2b), although mosquitoes may partly contribute to higher *C*_t_ values in vectors. NHPs also had higher median RPM compared with mosquitoes (median RPM = 5.88, range: 2.99–6.68, *n* = 89 vs median RPM = 5.04, range: 4.50–5.45, *n* = 9; Wilcoxon rank-sum *P* = 0.003). Mosquito pools showed higher N_50_ values (732 bp, range: 331–945) than NHP tissues (448 bp, range: 201–585; Wilcoxon rank-sum *P* = 9.1 × 10^−4^; Fig. 2b and Supplementary Table 8), indicating longer viral fragments in vector libraries.

### YFV sequencing performance across NHP carcass decomposition stages

Current epizootic investigation guidelines in Brazil recommend sampling NHPs within 24 h of death^24^. At PEAL, reporting delays were short and right skewed, with most carcasses reported and sampled within 0–2 days (early-stage, intact/alive) and with progressively fewer reports at ≥3 days (later-stage, medium/advanced decomposition) (Fig. 2c). *C*_t_, N_50_ and RPM did not differ significantly between NHPs sampled as intact/alive (*n* = 34) and those in medium or advanced decomposition (*n* = 12; Fig. 2d); decomposition stage was unavailable for 43 NHPs (Supplementary Table 8). Coverage was similarly high across stages (early-stage: median 99.90%, range 27.54–99.91%; later-stage: median 99.90%, range 0.96–99.91%), with >70% genome recovery in 94.12% of early-stage and 83.33% of later-stage samples (Fig. 2d).

### Phylogenetic and transmission dynamics

**Fig. 3:**
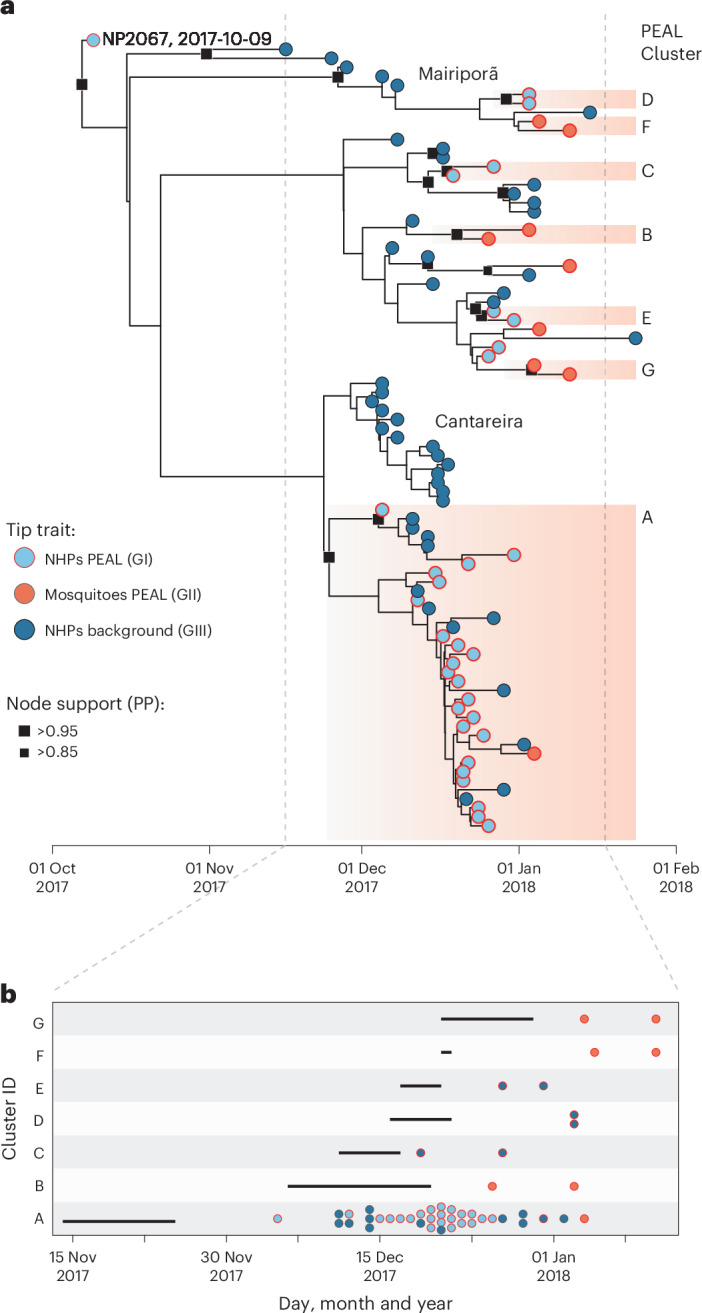
Temporal dynamics of YFV cluster introductions in PEAL State Park. **a**, Time-calibrated phylogeny of PEAL including local background genomes (*n* = 88), spanning a 107-day sampling window. Tips are annotated by host (NHP or mosquito) and whether they were sampled inside the park (ring). Squares mark node support (posterior probability, PP; large >0.95, small >0.85). Orange boxes highlight the seven PEAL clusters (A–G). The grey vertical dashed lines correspond to the timeframe expanded in **b**. **b**, Seeding and growth of each PEAL cluster. For each cluster, the left endpoint of the solid horizontal line represents the median time of the parent node of the most recent common ancestor (TMRCA), while the right endpoint marks the median TMRCA, corresponding to the onset of sustained local transmission. Circles correspond to dated samples in each cluster (same tip annotations as in **a**).

Maximum likelihood (ML) and Bayesian phylogenetic analyses placed the PEAL genomes within the previously identified YFV_SP_^25^ clade. Across three datasets (Brazil-wide, *n* = 1,063, sampling interval 15.2 years; São Paulo, *n* = 450, 3.7 years; PEAL, *n* = 88 0.3 years), ML trees consistently identified a dominant PEAL cluster (hereafter named ‘Cluster A’) with strong support (bootstrap 89–98) (Fig. 3a, and Supplementary Figs. 5 and 6). Molecular clock analyses dated the emergence of YFV in São Paulo state to mid-May 2016 (95% BCI: Feb 2016–Jul 2016; Supplementary Fig. 6). Within PEAL, 18/23 NHP genomes were identical, and one mosquito genome (ID81) differed by a single nucleotide (Fig. 3a). Focusing on the PEAL dataset, which showed adequate temporal signal (root-to-tip divergence *R* = 0.39), the within-outbreak evolutionary rate was 3.7 × 10^−3^ substitutions per site per year (95% BCI: 9.6 × 10^−4^ to 7.3 × 10^−3^), similar to previous estimates^9^. This corresponds to ~11 days of waiting time between mutations (95% BCI: 4–20 days).

Across PEAL, we identified 7 epizootic clusters (2–36 sequences) and 5 singletons within a single transmission season (Fig. 3, Methods). Interestingly, the early NP2067 case was a singleton outside all clusters, demonstrating that this case did not contribute to onward transmission at PEAL. Given the strong time dependency of evolutionary rates across broader timescales (see Supplementary Fig. 7), dating analyses were based on the PEAL and background dataset. Cluster A was the earliest introduction (around 14–25 Nov 2017), dominated the outbreak (*n* = 36; 24 group II, 12 group III) and persisted for 40–51 days, whereas later introductions generated only small, shorter-lived clusters (Fig. 3b).

### Transmission modelling and key epidemiological parameters

**Fig. 4:**
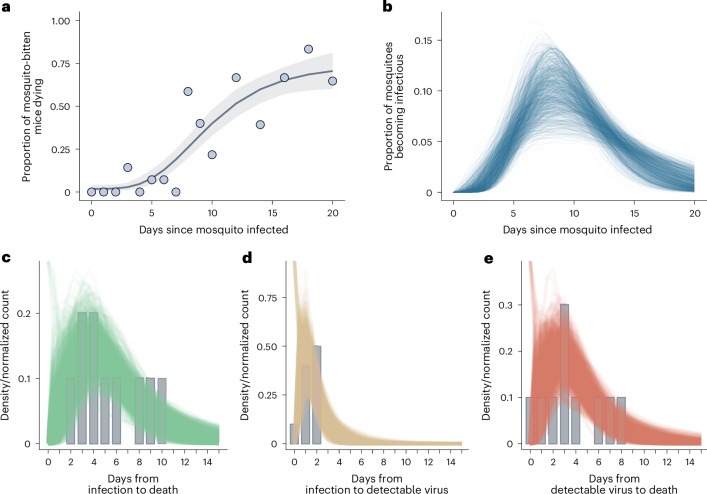
Empirical and estimated distributions informing key temporal parameters of YFV epidemic dynamics in a sylvatic cycle. **a**, Observed (points, data from ref. ^116^) and posterior predictive estimates (grey line and shaded ribbon show posterior median and 95% credible interval) of the proportion of mice that died following feeding by *H. leucocelaenus* mosquitoes inoculated with YFV; datapoints for days 1 and 2 were excluded since they probably reflected residual inoculum rather than a completed extrinsic incubation process. **b**, Estimated EIP of *H. leucocelaenus* mosquitoes based on data reported in ref. ^116^, with coloured lines representing draws from the posterior gamma distribution fitted to that data. **c**, Empirical and estimated distributions for the number of days from *A. guariba* exposure to YFV and subsequent death. **d**, Empirical and estimated distributions for the number of days from *A. guariba* exposure to YFV and becoming infectious. **e**, Empirical and estimated distributions for the number of days from *A. guariba* becoming infectious and death. In **c**, **d** and **e**, bars represent the empirical normalized frequencies based on data presented in ref. ^114^, and coloured lines represent draws from the posterior gamma distribution fitted to that data.

To characterize the transmission dynamics of the PEAL outbreak and assess the epidemic potential of YFV in NHPs, we developed an individual-based transmission model (IBM) that tracked each *A. guariba* at PEAL through infection, infectiousness and death, explicitly incorporating carcass-detection delays (Fig. 2c) and uncertainty in the extrinsic incubation period (EIP) of *H. leucocelaenus*. Five empirical distributions underline our framework: (1) delay from mosquito feeding to host death, capturing downstream effects of vector-mediated transmission (Fig. 4a); (2) EIP of *H. leucocelaenus* (median: 10 days, 95% CI: 8–12 days) (Fig. 4b); (3) delay from *A. guariba* death to carcass detection (median: ~2.1 days, 95% CI: 1.5–3.0 days) (Fig. 4c); (4) intrinsic incubation period (IIP) in *A. guariba* (median: 5.2 days, 95% CI: 4.3–6.2 days) (Fig. 4d); and (5) *A. guariba* infectious period (time from detectable viremia until death) (median: 3.8 days, 95% CI: 3.0–4.5 days) (Fig. 4e). The model parameterization and data sources are detailed in Extended Data Table 1.

### Estimation of reproduction numbers and outbreak dynamics

To estimate the basic reproduction (*R*_0_) and characterize the transmission dynamics of the PEAL outbreak, we calibrated our IBM using phylogenetic data, anchoring Clade A’s introduction around 19 Nov 2017 and accounting for the 5-week absence of confirmed cases after NP2067 (Fig. 1b). This allowed us to constrain the outbreak start and focus on the period of sustained transmission.

**Fig. 5:**
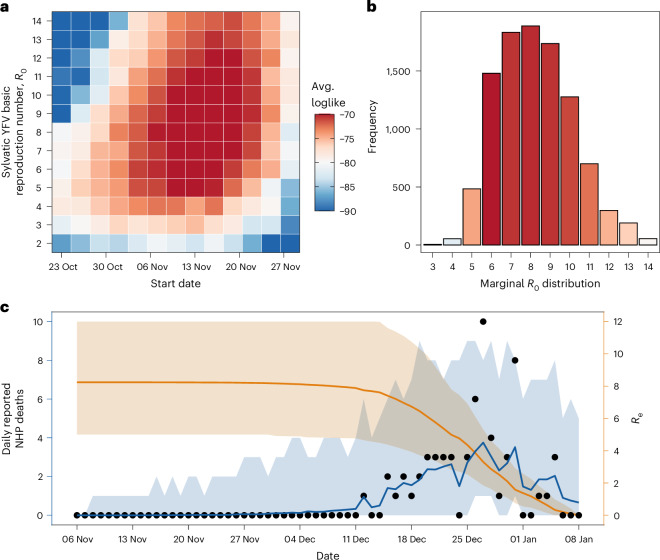
Transmission dynamics and epidemic potential of sylvatic YFV in PEAL. **a**, Average log-likelihood (colour scale) for each joint combination of the basic reproduction number (*R*_0_, *y* axis) and outbreak start date (*x* axis). Warmer tones indicate parameter pairs with higher posterior support. **b**, Marginal posterior distribution of *R*_0_ integrating over all start-date hypotheses shown in **a**. Bar height denotes posterior sample frequency; colours match the log-likelihood scale used in **a**. **c**, Model fit to daily reported non-human primate deaths (black circles, left axis) and the corresponding effective reproduction number over time (*R*_e_, orange line, right axis). Blue line and shaded ribbon give the posterior median and 95% credible interval for model-predicted deaths; the orange ribbon shows the 95% credible interval for *R*_e_.

Integrating phylogenies with epidemiological modelling yielded *R*_0_ = 8.2 (95% CI: 5.1–12.2) (Fig. 5a,b). This estimate is higher than estimates for urban outbreaks^26,27^, but falls within the range reported for Brazilian sylvatic settings^28^. Our posterior exploration across a two-dimensional grid of outbreak starting dates and reproduction numbers identified high-likelihood regions centred on introductions in the first weeks of November, with values typically above 6 (Fig. 5a). Our results were robust to alternative outbreak start dates, including scenarios with later epidemic onset (Supplementary Fig. 8). Because vectors are not modelled explicitly, *R*_0_ represents the product of the number of vectors infected by infectious *A. guariba* and the number of *A. guariba* subsequently infected by infectious vectors (that is, it integrates both vector and NHP transmission).

The posterior trajectories of the time-varying effective reproduction number, *R*_e_, which accounts for depletion of susceptible hosts, show a rapid decline in transmission over the course of the epidemic (Fig. 5c). This decline is consistent with the extensive infection and mortality that ultimately resulted in local extinction of *A. guariba* at PEAL.

### Repeating seeding but rare take-off of viral introductions

To assess the impact of different viral introductions, we used a branching-process model incorporating the IIP in *A. guariba* and EIP in *H. leucocelaenus*^29^. For Clade A (23/32, 71.9% of PEAL’s sequenced NHP cases), an estimated 1.5 host–vector–host generations (95% CI: 1–3) were required to generate the observed outbreak from a single infected mosquito. In contrast, the three minor introductions (6/32, 18.1% of sequenced cases) each contributed minimally, producing <0.4 generations of onward transmission.

## Discussion

We integrated ground and canopy entomology, systematic NHP surveillance, viral metagenomics and phylodynamics with transmission modelling to describe a sylvatic yellow fever outbreak at the forest–urban interface. Despite multiple introductions into PEAL, transmission was dominated by a single lineage introduced during a period of high *H. leucocelaenus* abundance, leading to rapid local extinction of *A. guariba*. Limited genetic divergence within this lineage and a fast within-outbreak evolutionary rate indicate a brief, intense transmission chain. Our modelling yielded an *R*_0_ ≈ 8.2 (95% CI 5.1–12.2), substantially higher than estimates from *Aedes*-mediated urban outbreaks^26,27^.

Temperature was the strongest correlate of *H. leucocelaenus* abundance, consistent with effects on development, survival and biting frequency^30^, while rainfall added little explanatory power due to collinearity (Supplementary Fig. 2). The multibite, multihost feeding behaviour across vertical strata of *Haemagogus*^31^ aligns with our detection of YFV-positive *H. leucocelaenus* at ground level. Higher YFV viremia in *H. leucocelaenus* compared with other vectors, together with high MIRs at PEAL (among the highest reported for the Atlantic Forest^5,32^) (Extended Data Fig. 2) and high *A. guariba* density^19^, probably accelerated NHP decline. Other primate taxa (*Callithrix*, *Sapajus*) showed no deaths or evident infections, consistent with lower susceptibility in shared habitats^33^. After *A. guariba* became locally extinct, vector infection rates quickly declined, and we found no evidence for onward YFV circulation despite reports of vertical transmission in *H. leucocelaenus*^34^ and the presence of *A. albopictus* and *A*. *aegypti* at PEAL.

Metagenomic sequencing provided several advantages during the investigation. It outperformed tiled-amplicon sequencing, resolved vector and host identities, and revealed an unexpected HAV co-infection in *A. guariba*. RPM tracked viral load and may serve as a surrogate when RT–PCRs are unavailable, or when molecular diagnostic performance is affected by primer–template mismatches. Recovery of YFV genomes from carcasses ≥3 days post mortem indicates that sampling windows could be extended to reduce missed epizootics. These results also reinforce the need to update tiled-amplicon schemes with contemporaneous genomes, as is standard for SARS-CoV-2 (ref. ^35^).

Ecological context and stochasticity determined whether introductions resulted in sustained transmission or self-limited events. The dominant cluster probably arose through founder effects and ecological bottlenecks, with lineage fitness shaped by local host–vector interactions^36^. This aligns with reservoir–host spillover theory, which predicts frequent fade-outs unless ecological and epidemiological conditions support onward transmission^17^. NP2067 was a self-limited event, occurring in a sector of PEAL with no detectable *H. leucocelaenus* during thermally unfavourable conditions (Fig. 1c). In contrast, later introductions during warmer periods and higher vector abundance generated several distinct transmission clusters. Given the absence of earlier cases in Greater São Paulo, short-range primate movement is unlikely to explain NP2067. Long-distance seeding into PEAL and elsewhere^37,38^—via asymptomatic carriers or viraemic individuals^6^, transported vectors or NHPs^39^, or windborne dispersal of infected mosquitoes^40^—remains plausible. *H. leucocelaenus* live for ~21 days and can disperse >5 km (refs. ^41,42^), and wind speeds up to 6.5 m s^−1^ during the outbreak could have transported mosquitoes tens of kilometres within hours^43^, well within ~25 km of earlier foci (Atibaia, Jundiaí). Purpose-built aerial sampling^44^ could help determine the contribution of windborne movement of YFV-competent mosquitoes.

Recent YFV resurgence near urban areas in 2024/2025 highlights the need for sustained NHP and vector surveillance, even after apparent fade-outs. At forest–urban boundaries, where spillover risk is elevated, viral metagenomics could detect YFV and other arboviruses (for example, St Louis encephalitis virus, Mayaro virus^45–47^) while providing insight into host and vector feeding patterns^48^. Detection of YFV in *Callithrix* in urban Minas Gerais further underscores ongoing spillover risk in densely populated areas of southeast Brazil^49^. With expanding *Callithrix* populations, proximity to the Cantareira Forest (PEC) and São Paulo metropolis, and with >1.6 million annual visitors, PEAL remains a high-risk area for YFV.

These considerations also have implications for the conservation and management of susceptible primates. *A. guariba* populations in Atlantic Forest fragments are endangered^50^, and immunization strategies for selectred NHPs (including golden-lion and golden-headed lion tamarins^51^) may warrant evaluation^52^. Under conditions similar to PEAL (*R*_0_ ≈ 8.2) and assuming 99% vaccine efficacy, ~89% coverage would be required to prevent outbreaks. This illustrates both the potential value and the ethical, ecological and logistical challenges of intervening in wild primate populations, including achieving high coverage in fragmented habitats and engaging local communities and stakeholders.

Our study has several limitations. The host-death delay distribution was derived from mouse data and may differ in NHPs. We assumed that mid-November cases reflected a single introduction, although this clade comprises about two-thirds of sequenced NHP PEAL genomes. Partial carcass recovery and modest sample sizes may bias cluster inference, although cross-dataset concordance mitigated this. Gaps in mosquito sampling, particularly at the canopy level, highlight the need for automated trapping with integrated meteorological logging^53^. Moreover, the absence of IgM serology and HAV immunohistochemistry prevented confirmation of HAV infection or exposure. Finally, *R*_0_ estimates are sensitive to ecological variation in primate density, contact rates and *Haemagogus* abundance, which can vary widely; previous studies report wide variation in *R*_0_ globally^26^ and within Brazil (2.7–7.2)^28^. Comparative, multisite studies integrating ecological, behavioural and genomic data are needed, particularly in light of the recent 2024–2025 outbreaks^54–56^.

Addressing these gaps will require complementary and scalable monitoring approaches. Low-cost and field‑deployable diagnostics^57^, community‑based carcass reporting systems (for example, SISS‑Geo^58^) and non‑invasive monitoring tools—including improved canopy-level entomological surveillance^59^, areal-imagery and acoustic tracking of NHPs^60^, and the use of NHP saliva and mosquito‑blood‑meal analyses^48^—could together accelerate outbreak detection and response. These approaches could also refine estimates of NHP density and help assess susceptibility and exposure across multiple primate families, both at urban–forest edges in the Atlantic Forest and in undersampled biomes such as the Amazon Forest and inland Cerrado where YFV persists in an enzootic cycle^61^.

Overall, our findings show that explosive yellow fever outbreaks at the forest–urban interface arise from interactions between vector ecology, host susceptibility and environmental drivers. With YFV resurging across the Americas^56^, sustained multisource collaborative surveillance, rapid public health action and alignment with WHO’s elimination strategy remain essential to protect at-risk human and wildlife populations. Sentinel NHP deaths occurring alongside rising *Haemagogus* abundance mark a brief window for intervention, with transmission able to intensify within only a few host–mosquito–host generations. Keeping detection‑to‑action intervals as short as possible will therefore be critical for future yellow fever mitigation strategies at the forest–urban interface.

## Methods

### Ethics approval

Only naturally deceased NHP carcasses were sampled for YFV surveillance. The surveillance protocol for dead NHPs was approved by the Ethics Committee for the use of Animals in Research, Instituto Adolfo Lutz (nos. 0135D/2012 and 020 G/2014), and includes work in protected environmental areas.

### Study site

Parque Estadual Alberto Löfgren (PEAL; 186 hectares, 40% public use) in an Atlantic Forest fragment in northern São Paulo municipality (775–850 m elevation) contiguous with Cantareira State Park (PEC). It sits within São Paulo Green Belt Biosphere Reserve (UNESCO), part of the Atlantic Forest Biosphere Reserve. Public-use sectors include Horto Florestal, Olaria, Polo Ecocultural and Arboreto Vila Amália, which also contains ~256 residential dwellings and staff housing. Annual visitation exceeded 1.6 million in 2019. For spatial analyses, we grouped management sectors into three zones (A–C, Fig. 1a).

### Entomological surveys

Following YFV confirmation at PEAL, we conducted targeted mosquito surveys in 43 days from 22 Dec 2017 to 02 Oct 2018 (40 epidemiological weeks) at 39 points near NHP carcasses. Using personal protective equipment, teams applied a standard protocol with: (1) hand nets and (2) Nasci vacuum aspirators at ground level and (3) CDC light traps baited with CO_2_ (dry ice) at ground and canopy levels. Traps operated from 09:00 to 15:00 and aspirator collections were performed for a fixed duration per point within the same period. To capture mosquito–host interface zones, forest edges, peridomestic areas and forest trails were sampled.

Captured mosquitoes were frozen alive in liquid nitrogen and stored at −70 °C in labelled cryotubes. For each collection we recorded method, stratum (ground/canopy), coordinates, date, number of tubes and collection period. Specimens were identified on a cold table with standard taxonomic keys^62–64^. Females from the same place/date were pooled (1–10) by species (or genus if needed). Raw, georeferenced records are provided in Supplementary Table 1. Temporal and spatial sampling layouts, genus composition and relative abundance are shown in Supplementary Fig. 1. Abundance summaries stratified by collection method and height are shown in Extended Data Fig. 1.

All statistical analyses, data wrangling and visualization were performed in R (v.4.3.2)^65^ using tidyverse, ggplot2, lubridate and related packages unless otherwise specified. Additional packages used for specialized tasks are cited at first mention.

### Meteorological data for São Paulo municipality

Hourly total precipitation (tp), 2-m air temperature (*T*) and 2-m dewpoint temperature (*d*) for 1 Jan 2017–31 Dec 2018 were retrieved from the ERA5-Land reanalysis dataset provided by Copernicus Climate Change Service (C3S) (0.1^0^ resolution)^66^. ERA5 temperatures (Kelvin) were converted to °C and precipitation (m) to mm. Relative humidity (rh) was computed using the Magnus approximation (parameters *a* = 17.625, *b* = 243.04; temperatures in °C):

1 $$\mathrm{rh}=100\times \exp (d\times a)/(d+b)\exp (T\times a)/(T+b)$$

ERA5-Land was overlaid on LandScan annual population rasters (1/120° resolution^67,68^) using geobr (v.1.8)^69^ municipality boundaries (majority-area tule when borders intersected a cell). Each LandScan cell inherited the nearest ERA5-Land grid-point values (nearest-neighbour mapping), yielding a per-cell table with administration unit, population and hourly meteorological variables for each pixel. Population-weighted hourly series were computed for São Paulo municipality and summarized into daily and then monthly indices (temperature minima/means/maxima; relative humidity minima/means/maxima; precipitation sum of daily totals) (Supplementary Table 4). These indices approximate meteorological conditions experienced across São Paulo municipality (including PEAL) during the study period.

### Association between vector abundance and climate factors

Monthly counts of *H. leucocelaenus* were modelled with negative-binomial GLMs. Covariates were *z* standardized, and predictors included contemporaneous and 1-month-lagged temperature (min/mean/max), relative humidity (min/mean/max) and cumulative rainfall. Unless specified, all hypotheses were two-sided and interpreted at alpha = 0.05. Model selection used the Akaike information criterion (AIC). Results are reported as rate ratios (RR) with two-sided 95% confidence intervals (CIs) and pseudo-*R*^2^. Collinearity (particularly between temperature and rainfall) (Supplementary Fig. 2) was assessed and, where indicated, addressed via interaction/quadratic terms. Models were fitted using MASS::glm.b, and correlations were summarized with corrplot^70^ and regression tables generated with stargazer^71^ (Supplementary Tables 5 and 6). An identical framework was applied to the four most frequent genera at PEAL (*Haemagogus*, *Aedes*, *Limatus* and *Culex*) (Supplementary Table 7). Coefficients are reported as RR per 1 °C and per unit rainfall, with 95% CIs and pseudo-*R*^2^.

### NHP carcass collection and notification

On Oct 9, 2017, the first confirmed YFV case at PEAL was detected in a southern brown howler monkey (*A. guariba*). During the ensuing outbreak, carcasses were collected under routine wildlife surveillance and samples were collected for virological testing. For each carcass, we recorded detection date, coordinates and decomposition stage (early-stage ≤48 h, medium/advanced >48 h post mortem) based on external morphology and soft-tissue preservation; no carcasses in desiccated/remains stages were collected (≥144 h post mortem) (Supplementary Table 10). Notifications were filed by the Wild Animal Management Center (CeMaCas) and reported to the São Paulo State Health Secretariat via Information System for Notifiable Diseases (SINAN), following Brazilian Ministry of Health guidelines^24^.

### Nucleic acid isolation and YFV RT–qPCR

Specimens were tested for YFV by RT–qPCR in accordance with the YFV Brazilian National Surveillance Program^24^. For NHPs, liver fragments were homogenized with magnetic beads (Magna Lyser, Roche), and RNA was extracted from 700 μl of homogenate (QIAamp RNA Blood mini kit, QIAGEN) following manufacturer instructions. Mosquito pools were homogenized in 700 μl phosphate-buffered saline (PBS, 0.75% bovine albumin, penicillin 100 U ml^−1^ and streptomycin 100 μg ml^−1^) using the MagNA Lyser (Roche) and RNA extracted (QIAamp Viral RNA mini kit, QIAGEN). Viral detection was conducted using the Go*Taq* 1-Step RT–qPCR (Promega) at Instituto Adolfo Lutz Virology Centre as previously described^72^, with standard RT–qPCR cycling conditions: reverse transcription (45 °C for 10 min), enzyme activation (95 °C for 10 min), followed by 40 cycles (95 °C for 15 s and 60 °C for 45 s for annealing/extension) on an ABI7500 Real-Time PCR (Thermo Fisher).

### Species‑specific *C*_t_ analysis across mosquito species

To compare viral RNA abundances across mosquito species, we analysed *C*_t_ values from YFV-positive pools of non-blood-fed females, combining 9 observations from this study with 109 observations from three Brazilian datasets^5,73,74^. Species names were harmonized (grouping *H. janthinomys* and *H. capricornii* as females are morphologically indistinguishable^75^); assay protocols^72,76,77^, date, municipality and pool size were recorded. We compared *H. leucocelaenus* against other species within each assay using two-sided Mann–Whitney/Wilcoxon tests with Benjamini–Hochberg FDR adjustment across pairs. To account for assay and pool size, we fitted a linear model with *C*_t_ ≈ pecies + assay + pool size (Extended Data Fig. 2). Statistical tests were conducted in R and results tidied with broom^78^. The source data (species, assay, pool size, pool-level *C*_t_, references) are provided as Supplementary Table 2.

### *H. leucocelaenus* YFV natural infection rates

We estimated mosquito infection rates using (1) minimum infection rate (MIR; per 1,000) and (2) a pooled-binomial maximum-likelihood approach accounting for variable pool sizes^79^. To contextualize PEAL, we compiled Brazilian studies testing ≥10 pools per species and extracted MIRs for *H. leucocelaenus* (*n* = 7), *H. janthinomys* (*n* = 5) and ‘other species’ (*n* = 4, including *Sabethes soperi, Sabethes chloropterus, Aedes scapularis* and *Aedes taeniorhynchus*)^5,32,80^ (Extended Data Fig. 3). Species-level MIR distributions were compared using Kruskal–Wallis followed by Benjamini–Hochberg FDR-adjusted two-sided pairwise Wilcoxon rank-sum tests. The source data (species, municipality, number of pools, total mosquitoes and pool sizes (when available), MIR, dates and references) are provided as Supplementary Table 3.

### Viral metagenomics

A schematic of laboratory and phylogenetic analyses is shown in Supplementary Fig. 4. YFV-positive NHP tissues and mosquito pools were sequenced with a validated SMART-9N metagenomic sequencing protocol^9,10,18^. Briefly, 44 μl of extracted viral RNA were DNase treated (TURBO DNase, Thermo Fisher), cleaned up and concentrated to 10 μl (RNA Clean & Concentrator-5, Zymo). Extraction blanks and no-template controls were included at each step to monitor contamination. MinION libraries were prepared from 50 ng of double-tagged cDNA per sample, barcoded and pooled equimolarly using the EXP-NBD104 (1–12) and EXP-NBD114 (13–24) Native Barcoding kits (Oxford Nanopore Technologies (ONT)), and sequenced with the SQK-LSK109 sequencing kit on fresh FLO-MIN106 (R.9.4.1) flowcells (ONT). Three 24-plex libraries were run on a GridION (ONT) under MinKNOW 1.15.1 (ONT) using a standard 48-h script.

### Tiled‑amplicon sequencing and cross‑method benchmarking

A subset of 56 YFV-positive NHP and mosquito samples was sequenced with a validated multiplex tiled-amplicon protocol (YFV500/V1)^81^ designed during the early stages of the outbreak (April 2017)^9^. Overlapping 500 bp amplicons spanning the coding region of the YFV South American genotype I outbreak clade were generated and sequenced on GridION (ONT). We compared *C*_t_ values with paired differences in consensus sequence coverage between tiled-amplicon vs viral metagenomics, fitting a linear model (stats v.3.6.2 package) to calculate the *C*_t_ at which the methods perform equivalently (Supplementary Fig. 3). Source data can be found in Supplementary Table 8. Coverage profiles and recurrent amplicon dropouts for the YFV500/V1 multiplex primer scheme are shown in Extended Data Fig. 4.

### Consensus genome generation

FASTQ files were demultiplexed and adapter trimmed (Guppy v.5.0.16, ONT), mapped to YFV South American genotype I (BeH655417; GenBank accession number JF912190) with minimap2 (v.2.28)^82^, then processed with SAMtools (v.1.20)^83^. Raw read counts and fragment N_50_ were summarized with NanoStat (v.1.1.2)^84^. Genome visualization, mapped-read counts and depth profiles were inspected with Tablet (v.1.17.08.17)^85^ and SAMtools (v.1.20)^83^. Variants were called using Medaka v.1.12.1 (ONT) and consensus sequences generated with margin*_*cons Medaka v.1.12.1 (ONT); regions with <10× depth were masked. Sequencing metrics, *C*_t_ values and metadata are provided in Supplementary Tables 8 and 9.

### Host and vector identification and detection of other viral pathogens from metagenomic sequencing data

Despite DNAse treatment to remove background DNA in the SMART-9N protocol, sufficient host and vector reads remained to confirm species. Demultiplexed FASTQs were mapped with minimap2 (v.2.28)^82^ to *H. leucocelaenus* (GenBank accession number NC057212.1*)* and *A. guariba* (GenBank accession number NC_064186.1). Alignments were processed with SAMtools (v.1.20)^83^ and consensus fragments were generated as described above. Species identities were verified using BLASTn v.216.0 against NCBI GenBank^86^. To screen for non-YFV viruses, reads were classified with Kraken2 (v.2.1.3)^87^ (RefSeq complete viral genomes^88^), inspected in pavian^89^ and validated by reference mapping (NCBI Viral Genome Resource^90^). We considered a detection as corroborated when mapping showed coherent coverage across the expected genomic region (breadth and depth consistent with library yield) and the signal was absent from concurrent negative controls.

### HAV consensus and phylogeny

Reads from NP2754 were mapped to GenBank accession number MG049743.1 and a consensus was generated using the same pipeline (positions with depth <10× masked). Genotype assignment used the Hepatitis A virus genotyping tool (v.1.0)^91^. For context, we retrieved the closest 100 sequences to NP2754 HAV via BLASTn (v.216.0)^86^ and NCBI Virus (Brazilian sequences >500 bp, Oct 2025)^92^. After excluding outliers with very long branch lengths (AF268396.1, MG181943.1, MZ557007.1) and removing records without country information, sequences were aligned with MAFFT v.7 (–auto) and end trimmed to the longest region shared with the NP2754 HAV consensus. After additional QC with TempEst (v.1.5.3)^93^, a 97-sequence alignment (6,638 bp, sampling range = 68 years) was used to estimate a maximum-likelihood tree using IQ-TREE2 (v.2.3.66)^94^ with ModelFinder Plus^95^ and node support from UFBoot2 (1,000) and SH-aLRT (1,000) replicates^96^. The final tree was midpoint rooted and is shown in Extended Data Fig. 5.

### YFV nucleotide data collation, curation and ML phylogenies

Public YFV sequences were retrieved from GenBank and filtered to include Brazil-origin samples with >70% genome coverage (based on submitter metadata or computed coverage) and exclude laboratory strains, chimaeras and records lacking a verifiable collection date/location. For samples represented by multiple assemblies, we kept the highest coverage or earliest submission and removed duplicates. PEAL sequences from this study were added after the same QC. Coding regions were aligned with MAFFT v.7 (–auto)^97^ and manually curated in AliView (v.1.28)^98^. Sequences with >30% ambiguous sites or obvious frame disruptions were excluded.

From the curated alignment (10,221 bp) we defined three datasets: (1) Brazil (2008–2023; *n* = 1,063, 13 states, including PEAL; Supplementary Fig. 5); (2) São Paulo (a subset focused on the previously described YFV_SP_ clade^25^, including sequences from Goiás, Minas Gerais, São Paulo and Rio de Janeiro collected between 2015–2019; *n* = 450, including PEAL; Supplementary Fig. 6); and (3) PEAL (*n* = 88 near-complete and complete PEAL genomes from groups I–III; Fig. 3a). Per-sequence QC metrics and accession numbers of the PEAL dataset are provided in Supplementary Table 9. We inferred ML phylogenies using IQ-TREE (v.2.2.2.6)^94^ with the ModelFinder Plus model selection^95^ and 1,000 ultrafast bootstrap replicates^96^. Clock-like signal was assessed with TempEst (v.1.5.3)^93^. For the PEAL dataset, we additionally applied Bayesian Evaluation of Temporal Signal (BETS)^99^, computing four independent analyses: strict vs relaxed molecular clock (with log-normally distributed rate variation among branches^100^), and exponential vs Bayesian skygrid tree priors^101^. Including sampling dates in BETS provided strong support for the presence of temporal signal in the PEAL dataset (log Bayes factors > 18) when including sampling dates vs the null model (no sampling dates) in all four clock and tree prior model combinations. Moreover, the relaxed clock with a skygrid tree prior outperformed the relaxed clock with an exponential tree prior (log Bayes factors = 15.5). Alignments, trees and XMLs are provided in Data availability.

### Bayesian phylogenetic inference

Time-scaled phylogenies for the São Paulo and PEAL datasets were inferred using BEAST X^102^ with BEAGLE v.3 acceleration^103^ under an autocorrelated relaxed clock (log‑normal among‑branch rate variation^100^), Bayesian skygrid demographic model (using 47 grid points and cut-off of 4 years for the São Paulo dataset, and 35 grid points and a cut-off of 3 years for the PEAL dataset)^101^, HKY substitution with among‑site heterogeneity^104,105^ and a CTMC reference prior on the clock rate^106^. Tip dates were set at the reported precision (day, month or year). Two independent MCMC chains (100 million steps, sampling every 10,000 steps) were run per dataset. Convergence and mixing of the MCMC chains were assessed in Tracer (v.1.7.2)^107^ (effective sample size > 200 for all key parameters and visual agreement across duplicate runs). After 10–25% burn-in, runs were combined (LogCombiner v.1.10.5) and summarized as maximum credibility (MCC) trees (TreeAnnotator (v.1.10.5)^108^). Time-dependence of evolutionary rates in YFV South American genotype 1 was explored by comparing posterior rates inferred from the PEAL and São Paulo datasets with published data^9,109–112^ (Supplementary Fig. 7).

### Epizootic cluster definition

On PEAL phylogenies, a cluster was defined as a monophyletic group of ≥2 PEAL genomes (NHP or mosquito) with strong support (ML bootstrap >90 and/or posterior probability >0.90). We verified monophyly in the São Paulo and Brazil‑wide ML trees; all remained strongly supported (bootstrap >95) except Cluster D (2 *H. leucocelaenus* sequences), which we retained on the basis of support within the PEAL analysis (bootstrap = 81). Given the observation that YFV evolutionary rates can be time dependent (Supplementary Fig. 7), cluster dating used the single-season PEAL dataset (root-to-tip *r* = 0.39). For each cluster we recorded the (1) median date of the cluster most recent common ancestor (MRCA); (2) median date of the parent of the MRCA; (3) midpoint between (1) and (2); and (4) cluster duration, defined as the number of days from the last sampling date to (3) (Fig. 4b).

### Modelling YFV transmission dynamics

#### Model framework

We simulated the PEAL epizootic in *A. guariba* using an IBM implemented in R (package ‘individual’ (v.1.1.17)^113^). Each NHP transitions through susceptible (S), exposed (E), infectious (I) and dead (D) states, assuming 100% lethality in *A. guariba* at PEAL (approximating observed lethality; Fig. 1b). Upon death, individuals are detected and sampled with sampling probability *p*_obs_, reflecting incomplete ascertainment of all deceased *A. guariba*. A summary of the model parameterization and sources for the parameters can be found in Extended Data Table 1.

#### The infection process and the force of infection (FOI)

We assumed density-dependent transmission, such that each susceptible *A. guariba* experiences a force of infection (FOI) proportional to the number of infectious individuals at time *t*, *I*(*t*). Thus:

2 $$\lambda (t)=\beta I(t)$$

where *β* is the transmission rate. If *γ* is the average duration of infectiousness, the basic reproduction number *R*_0_ is:

3 $${R}_{0}=\beta S(t)\gamma$$

representing the total number of infected *A. guariba* that a single infected *A. guariba* infects during the course of their infection, in a population the size of the susceptible *A. guariba* population in PEAL. Because the model does not explicitly represent mosquito populations, *R*_0_ reflects the combined host–vector–host process (that is, the product of the number of vectors infected by an infectious *A. guariba* and the number of *A. guariba* subsequently infected by infectious vectors).

#### IIP in *A. guariba*

Following infection, individuals enter an exposed (E) state before becoming infectious. The time spent in the E state is drawn from a gamma distribution and reflects both the IIP of YFV in NHPs which was estimated from ref. ^114^ (Fig. 4c,d) and the EIP in the vector (see below). In the case of the incubation period of YFV in *A. guariba*, a gamma distribution was fitted to this data using the Stan (v.2.36.0) probabilistic programming language^115^. Weakly informative gamma priors were placed on both the shape (*a*) and scale (*b*) parameters of the gamma distribution for the IIP, as follows: *a* ≈ Gamma(1, 0.5); *b* ≈ Gamma(1, 2). The model was run with 4 chains of 2,000 iterations each, of which the first 1,000 iterations were used as warm up for adaptation, resulting in a total of 4,000 posterior samples. Convergence was confirmed by standard diagnostics (*R*-hat < 1.02).

#### EIP in *H. leucocelaenus*

We reanalysed the experimental data of ref. ^116^ (Fig. 4a,b) to estimate the EIP for *H. leucocelaenus*. Briefly, *Haemagogus* mosquitoes were inoculated with YFV and then made to feed on mice some number of days following inoculation. The proportion of mice that go on to die following *Haemagogus* feeding is therefore indicative of the proportion of mosquitoes that have completed the EIP and are thus able to successfully transmit YFV upon feeding. A gamma distribution was fitted to the data presented in the paper for the 25 °C experiment (representing the most complete experiment that is nearest to the temperatures measured in PEAL as shown in Fig. 1c). Uniform, minimally informative priors were placed on both the shape (*a*) and scale (*b*) parameters of the gamma distribution, with each specified as uniform(0.1, 10). We excluded the results associated with days 0 and 1, which are associated with high mortality and probably represent infection occurring from residual viable YFV from injection into the vector, rather than new infectious YFV virions generated by replicative cycles inside the vector. As above, the model was run with 4 chains of 2,000 iterations each, of which the first 1,000 iterations were used as warm up for adaptation, resulting in a total of 4,000 posterior samples, and convergence was confirmed across chains (*R*-hat < 1.01). Although vectors were not explicitly modelled, this empirically derived EIP was included in the delays governing host–vector–host transmission, ensuring that expected delays between an *A. guariba* becoming infected and (indirectly, through infectious *Haemagogus* vectors) generating subsequent infections in other NHPs were accurately captured. Following this intrinsic and extrinsic incubation period, the infected *A. guariba* were then presumed to begin contributing to onward transmission (that is, the FOI term described above).

#### NHP infectious period, time to death and observation process

We assumed that YFV is 100% lethal in *A. guariba* and thus, once infectious, an individual NHP eventually goes on to die due to YFV. In our IBM, this delay between infection and death was drawn from a gamma distribution, the parameters of which were estimated from reanalysis of experimental *A. guariba* infection data presented in ref. ^114^. As previously, gamma distributions were fitted to this data using the Stan (v.2.36.0) probabilistic programming language. Weakly informative gamma priors were placed on both the shape (*a*) and scale (*b*) parameters of the gamma distribution, as follows: *a* ≈ Gamma(1, 0.5); *b* ≈ Gamma(1, 2). The model was run with 4 chains of 2,000 iterations each, of which the first 1,000 iterations were used as warm up for adaptation, resulting in a total of 4,000 posterior samples. Convergence of the chains was confirmed (*R*-hat < 1.02).

Upon death, each NHP was reported with probability *p*_obs_, which represents the probability that a dead NHP was observed, and a notification sent that enabled enumeration and sampling to occur. In practice, we implemented this by drawing a Bernoulli random variable at the time of death for each individual, with parameter *p*_obs_ representing the chance the death was recorded:

4 $${D}_{\mathrm{reported}} \sim \mathrm{Binomial}({D}_{\mathrm{obs}},{p}_{\mathrm{obs}})$$

Given confirmation of local extinction of the NHP population of PEAL (see also Fig. 1b), this probability was estimated empirically from the collated data for PEAL, specifically by dividing the number of enumerated *A. guariba* deaths over the modelled time period by the total *A. guariba* population of PEAL immediately preceding the modelled time period. Detection and reporting of deaths occurred with some delay following death; this delay was estimated empirically from local veterinarian team estimates of the stage of corpse decomposition at detection (that is, the number of days since the NHP had died, see also Supplementary Table 8), and its estimation is described in further detail below. Specifically, a mixture of exponential and gamma distributions was fitted to the data using the Stan (v.2.36.0) probabilistic programming language^115^. Weakly informative gamma priors were placed on both the shape (*a*) and scale (*b*) parameters of the gamma distribution as follows: *a* ≈ Gamma(1, 1); *b* ≈ Gamma(2, 2). For the exponential distribution, we placed a uniform(0.1, 10) prior on the rate parameter. As above, the model was run with 4 chains of 2,000 iterations each, of which the first 1,000 iterations were used as warm up for adaptation, resulting in a total of 4,000 posterior samples. Convergence of the chains was confirmed (*R*-hat < 1.02). See Data availability for full details of the IBM and its implementation. The empirical and estimated distributions for the delay between *A. guariba* death and that death being reported and enumerated are shown in Fig. 2c.

#### Inference of *R*_0_ and fitting to PEAL timeseries data

To infer the basic reproduction number (*R*_0_) of YFV-infected *A. guariba* in PEAL during the outbreak, we fitted the IBM to the timeseries of NHP YFV deaths in PEAL. We placed an informative prior on the timing and intensity of importations based on the dated phylogenetic analysis of YFV PEAL dataset (see Fig. 3), constraining possible start dates using the posterior distribution of the parental node of Cluster A (sensitivity analyses using the midpoint of Clade A’s parental node and the MRCA of Clade A as the basis for the prior on the start data are shown in Supplementary Fig. 8). Because the first detected case (NP2067) was phylogenetically unrelated to subsequent transmission (a scenario that was consistent in all three phylogenetic datasets) and to the subsequent sustained outbreak that led to local extinction, we excluded deaths before 15 Nov 2017 (Fig. 1b) from model fitting, treating this early event as a separate, self‑limited introduction.

Daily incidence of reported NHP deaths *y*(*t*) were modelled as the Poisson realizations of the expected number of detected deaths, *D*_reported_(*t*), reported on day *t*:

5 $$Y(t) \sim \mathrm{Poisson}({D}_{\mathrm{reported}}(t))$$

The likelihood for the entire timeseries is therefore given by:

6 $$\mathrm{Poisson}(Y(t){|}_{D\mathrm{reported}}(t))$$

A closed-form expression of the likelihood of the observed data given the model and its parameters was not analytically tractable, so we used particle filtering methods to obtain unbiased likelihood estimates^117^. To generate an estimate of the marginal model likelihood for each parameter combination, we conducted a parameter scan across different parameter combinations, utilizing a bootstrap particle filter with 250 particles. If the expected values of count distributions derived from the modelling framework are equal to 0 when empirically observed data are not 0, this results in particles of 0 weight, which can lead to the particle filter estimating the marginal likelihood to be 0 for all particles, and prevent the bootstrap particle filter from sampling efficiently or appropriately. To mitigate this, we followed the approach of ref. ^118^ and added a small but non-zero weight for each particle at every observation. This was achieved by adding a small amount of noise (exponentially distributed with mean 10^−2^) to modelled incident death values of 0. For each parameter combination, we ran 10 independent replicates with a bootstrap particle filter (to generate 10 independent estimates of the model likelihood for that parameter combination) and calculated the mean likelihood from these independent replicates. Model fitting was carried out within a Bayesian framework, with weakly informative priors used for the *R*_0_ and epidemic start date. For the prior on *R*_0_, we compiled 11 field-based estimates of *R*_0_ reported in a recent review^26^. A truncated normal was fitted to the data reported in this review to construct a prior distribution for *R*_0_ (mean ≈ 4.6, s.d. ≈ 2.7). The prior on the epidemic start date was constructed on the basis of the PEAL molecular clock analysis. Specifically, the start date of the outbreak was bounded by Clade A’s MRCA posterior distribution, fitting a Weibull distribution to this posterior and using it as the prior in the transmission model inferential framework.

#### Transmission generations and epidemic amplification

To interpret cluster sizes in terms of underlying transmission dynamics, we simulated a negative-binomial branching process (offspring mean *R*, dispersion *k*) with combined generation time *G* = EIP (*H. leucocelaenus*) + EIP (*A. guariba*). This allowed us to summarize the number of host–mosquito–host generations required to reach each observed cluster size. For the dominant Cluster A, the inferred size was consistent with 1–3 generations. Full details of the inferential framework and its implementation are available in Data availability.

### Reporting summary

Further information on research design is available in the Nature Portfolio Reporting Summary linked to this article.

## Supplementary information

Supplementary Information Supplementary Figs. 1–8 and Supplementary Information References.

Reporting Summary

Supplementary Tables 1–11 **Supplementary Table 1. Metadata for entomological collections, including sampling details, species identification and YFV testing results**. This table summarizes all mosquito collections undertaken at PEAL during the study period, including collection date, GPS coordinates, sampling height, collection technique, pool identifier, taxonomic identification (genus, species and sex), number of mosquitoes per pool and YFV RT–qPCR result.**Table 2. Review of YFV** ***C***_t_ values and RT–qPCR assays reported for different mosquito species in the literature. This table compiles published YFV RT–PCR results across mosquito species and locations, including state, municipality, collection date, species, number of individuals tested, mean *C*_t_ value, study citation, DOIs, and the RT–PCR protocol and corresponding reference. Groups (I–III) correspond to classifications defined in Extended Data Fig. 7. *C*_t_, cycle threshold; No., number of mosquitoes in each pool. **Table** **3.** **Review of YFV minimum** **infection rates (MIR) and maximum-likelihood estimates (MLE) reported for mosquito species in the literature**. This table summarizes MIR and MLE values for YFV‑positive mosquito pools reported across published studies, including geographic region (state and municipality), species, number of pools tested, number of positive pools and total mosquitoes sampled. Collection periods, inclusion criteria and study DOIs are provided for reference. MIR and MLE are reproduced as originally reported in each publication. **Table 4. Monthly meteorological indicators from ERA5‑Land, including temperature, humidity and rainfall, with corresponding** ***Haemagogus*** **activity**. This table reports monthly summary statistics derived from ERA5‑Land for São Paulo municipality, including minimum, mean and maximum temperature; minimum, mean and maximum relative humidity; and monthly cumulative rainfall. The table also includes the number of *Haemagogus* mosquitoes collected and the number of YFV‑positive pools detected in each month. **Table 5.** **Univariate negative-binomial models** **of** ***H. leucocelaenus*** **abundance** **and individual lagged meteorological variables**. Each column reports a separate univariate model relating monthly *H. leucocelaenus* catch to a single meteorological variable (temperature, relative humidity or cumulative rainfall) recorded 1 month before trapping. Coefficient estimates are shown with standard errors in parentheses. Pseudo‑*R*², log‑likelihood, the overdispersion parameter (*θ*) and AIC are reported at the bottom. Significance levels: *P* < 0.1; *P* < 0.05; *P* <0.01.    **Table 6. Multivariate negative-binomial models of** ***H. leucocelaenus*** **abundance and meteorological variables**. This table presents parameter estimates from multivariate negative‑binomial models evaluating monthly *H. leucocelaenus* abundance in relation to combinations of meteorological variables recorded 1 month before trapping. Coefficients are reported with standard errors in parentheses. Model fit metrics (pseudo‑*R*², log‑likelihood, dispersion parameter *θ* and AIC) are shown below each model. Significance levels: *P* < 0.1; *P* < 0.05; *P* < 0.01. **Table 7. Genus‑specific negative‑binomial models of monthly mosquito abundance at PEAL**. Negative‑binomial GLMs were fitted separately for each mosquito genus, with monthly counts as the dependent variable and mean temperature (°C) and cumulative rainfall (cm) included jointly as predictors. Results are presented as rate ratios (exp *β*) with two‑sided 95% confidence intervals and Wald *P* values; effects represent estimates conditional on the other covariate. Pseudo‑*R*² is defined as 1–(deviance/null deviance), and *N* indicates the number of months with observations. RR = rate ratio. **Table 8. Laboratory metadata and sequencing performance metrics for NHP and mosquito samples (Groups I–III).** This table compiles laboratory and sequencing information for all NHP and mosquito samples analysed in this study, including host species, epidemiological group, *C*_t_ value, decomposition stage and pool composition (for mosquitoes). Sequencing metrics include amplicon and metagenomic coverage, total reads, N_50_, mapped‑read counts, reads per million (RPM), percentage of mapped reads, average depth of coverage, percentage of genome covered at ≥10×, identity to the reference genome, and corresponding GenBank accession numbers. **Table 9. Metagenomic sequencing statistics for YFV genomes generated in this study**. This table summarizes sequencing statistics for the YFV genomes obtained through metagenomic (SMART‑9N) sequencing. Variables include sample group (I–III; see Extended Data Fig. 7), post‑mortem interval for NHPs, PEAL management zone, *C*_t_ value (where available), total and mapped reads, number of bases with ≥10× coverage, percentage of the YFV reference genome covered, and GenBank accession number. A dash (–) indicates data not available. PM, post‑mortem; No., number; bp, base pairs; Ref. (%), percentage of the reference genome covered. Additional metadata, including host species, coordinates, decomposition stage or pool size, are provided in Supplementary Table 2. **Table 10.** **NHP notification records, sequencing status, spatial metadata and date of death.** This table compiles all NHP records reported during the PEAL outbreak, including notification outcome, sequencing status, management zone, simplified zone classification, GenBank accession (where applicable), georeferenced coordinates and date of death (calendar date and epidemiological week). **Table 11. Virological and histopathological findings in NHPs by decomposition stage**. This table summarizes RT–PCR results, *C*_t_ values, immunohistochemistry (IHC) and major pathological findings for NHPs sampled at PEAL, stratified by carcass condition (intact/alive, medium or advanced decomposition ≥3days, and unknown).

## Data Availability

The YFV genomic data generated in this study are available in NCBI GenBank under accession numbers OQ714241–OQ714328. The near-complete hepatatis A virus (HAV) genome is available under accession number PV702359. Raw metagenomic sequencing reads (FASTQ files) have been deposited under the NCBI Sequence Read Archive (SRA) BioProject PRJNA1269522, with associated BioSample accessions SAMN48792130–SAMN48792218. Detailed laboratory protocols, including the viral metagenomic sequencing workflow and SMART‑9N primer sequences, are available at https://protocols.io ref. ^46^. The phylogenetic trees, genomic datasets and XML files are publicly accessible in our dedicated GitHub repository at https://github.com/CADDE-CENTRE/YFV_horto (ref. ^119^).

## Extended data

**Extended Data Table 1:** Description of the IBM model parameterisation and sources for model parameters

Parameter	Description	Reference
Transmission Rate (β)	Transmission rate parameter. Inferred directly from PEAL time-series data using particle filter-based likelihood estimation approach.	NA
Incubation period duration	Gamma distribution with shape X and rate Y giving average duration of Z days. Delay distribution exposed to infectious (that is the incubation period for YFV) in *A. guariba*. Estimated from analysis of data from Haemmert et al., 1950 (see Fig. 4c–e). Inverse of the expectation of this distribution is γ used in the calculation of R_0_	Laemmert et al.,^15^
Duration of infectiousness	Gamma distribution with shape X and rate Y giving average duration of Z days. Delay between *Alouatta* becoming infectious and dying from YFV. Estimated from analysis of data from Haemmert et al., 1950.	Laemmert et al.,^15^
EIP	Gamma distribution with shape X and rate Y giving average duration of Z days. Extrinsic incubation period that is the time taken for infected *Haemagogus* mosquitoes to become infectious. Estimated from analysis of data from Bates & Roca-Gacia, 1946 (see Fig. 4c–e). Gamma distribution with shape X and rate Y.	Bates & Roca-Garcia^16^à
Delay between death and observation	Exponential distribution rate parameter X. The delay between *A. guariba* death occurring, and that death being detected, reported and enumerated. This was estimated empirically from veterinarian estimated stage of corpse decomposition at detection (that is the number of days since the NP had died).	Empirically estimated
p_obs_	0.7625 (61 / 80) Probability of a dead *A. guariba* belonging to the PEAL population being detected, reported and successfully enumerated during the outbreak. Estimated empirically as the fraction of enumerated deaths relative to the overall known size of the *A. guariba* population in PEAL at the outbreak’s start.	Empirically estimated

## Code availability

The IBM YFV model is publicly accessible via GitHub at https://github.com/cadde-centre/YFV_horto (ref. ^119^), along with the scripts for the statistical analyses reported in this study. Tools, packages and software used in this study are publicly available.
