# Supplementary material for: Evolution and spillover dynamics of yellow fever at the forest–urban interface in Brazil
Source: Nat Microbiol. 2026 Mar 11;11(4):877–91. doi: 10.1038/s41564-026-02302-w (PMC13056586; doi:10.1038/s41564-026-02302-w)
Supplement: Supplementary file 1 — Supplementary Figs. 1–8 and Supplementary Information References. [file 41564_2026_2302_MOESM1_ESM.pdf]

nature microbiology

Supplementary information https://doi.org/10.1038/s41564-026-02302-w

Evolution and spillover dynamics of yellow fever at the forest–urban interface in Brazil

In the format provided by the authors and unedited

Supplementary Fig. 1

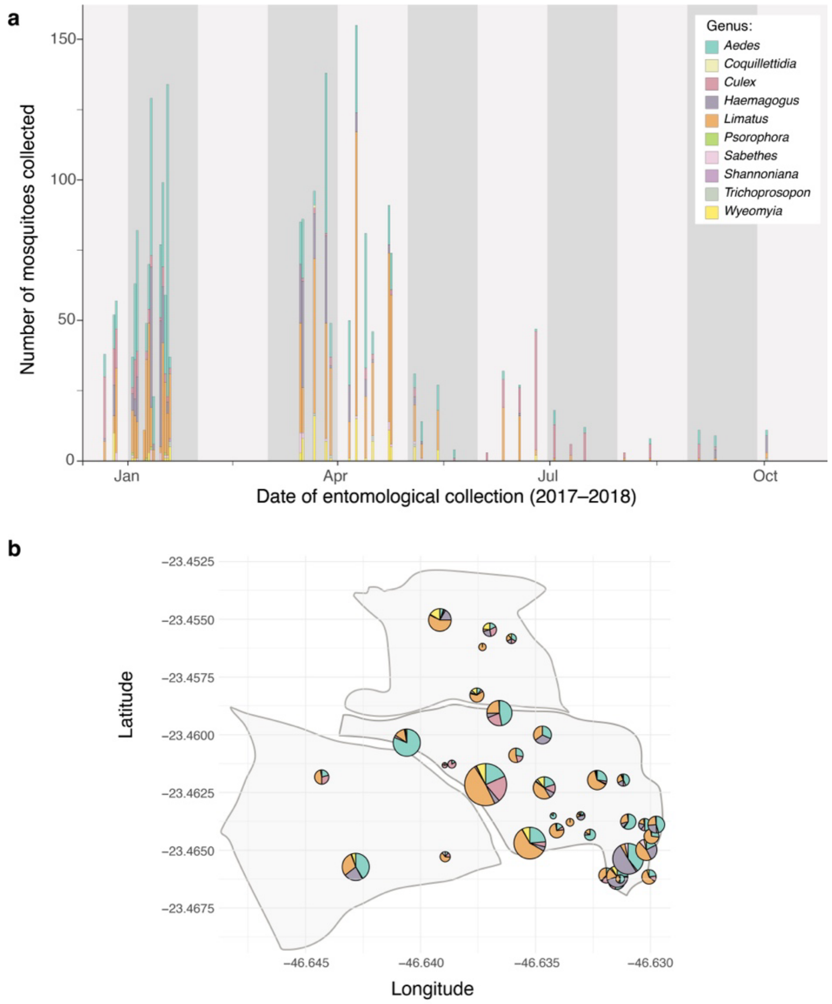

Spatial and temporal distribution of mosquito collections at PEAL. (**a**) Timeline of mosquito collections between December 2017 and October 2018, showing the total number of specimens collected per survey date, stratified by genus. (**b**) Georeferenced sampling sites across the park, with pie charts indicating the relative abundance of genera (coloured as in panel **a**) and circle size proportional to the total number of mosquitoes collected at each site. Raw data, including dates and coordinates of each location, are provided in **Supplementary Table 1**.

Supplementary Fig. 2

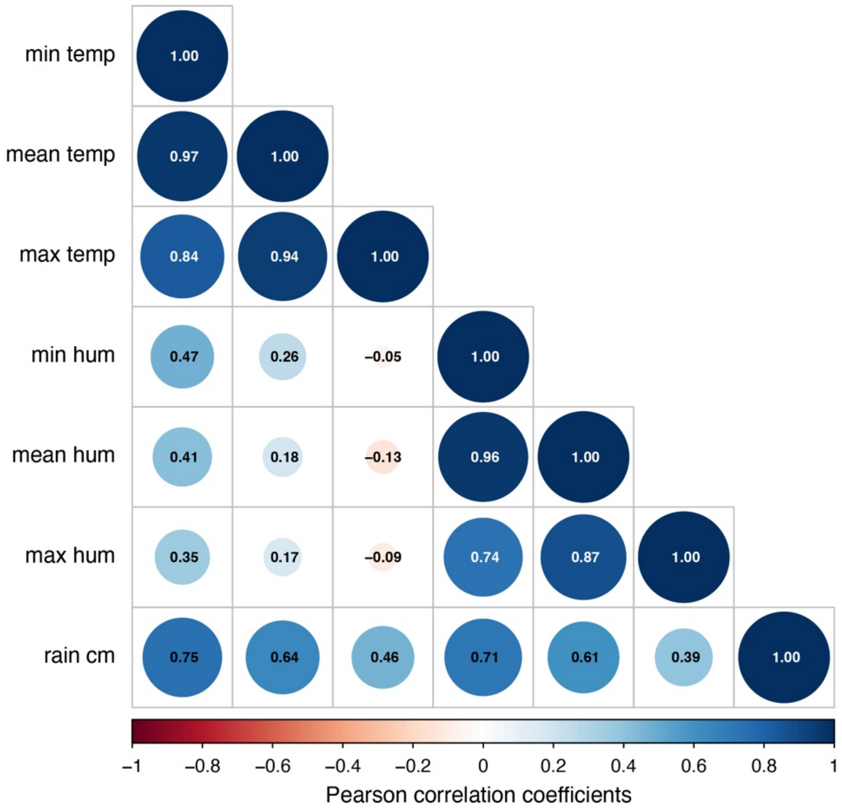

Pairwise correlations among monthly meteorological covariates used in the *Hg. leucocelanus* models. Pearson correlation coefficients between seven z-standardised climate variables (minimum, mean and maximum temperature; minimum, mean and maximum relative humidity; and cumulative rainfall) are shown as coloured circles. Circle area and colour intensity scale with correlation magnitude as shown on the right, with blue denoting positive and red denoting negative associations.

Supplementary Fig. 3

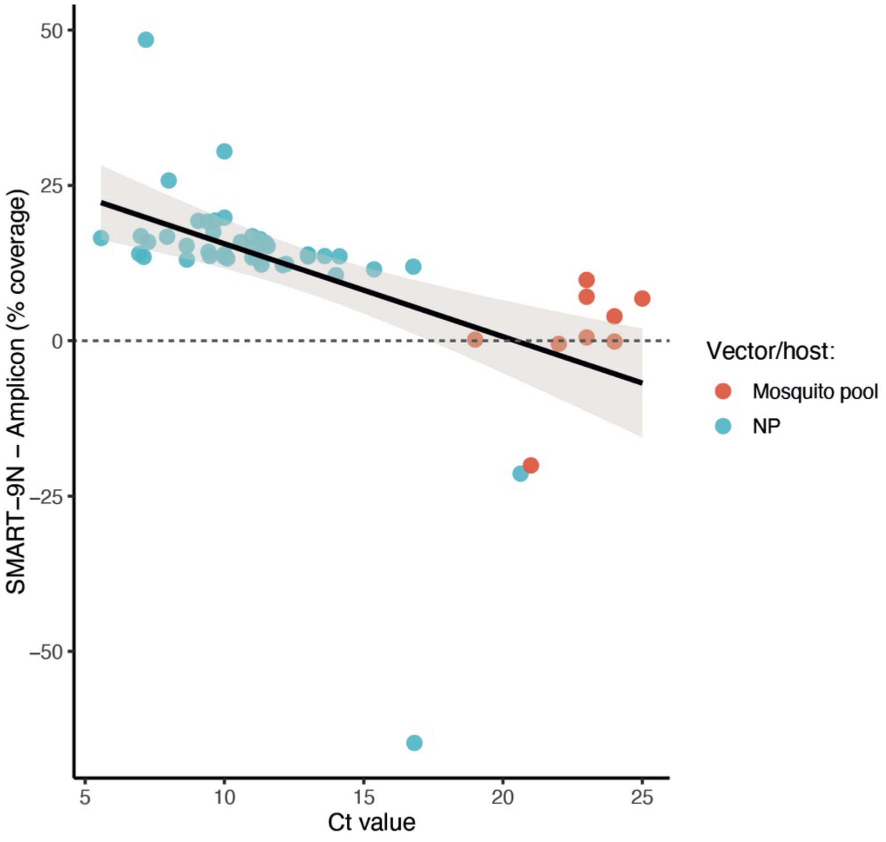

Relationship between RT-PCR Ct values and difference between consensus sequence coverage for paired samples sequenced with SMART-9N metagenomic sequencing and the tiled-amplicon scheme. Dots represent paired sequenced samples (blue = nonhuman primates, red = mosquitoes). The solid black line shaded band shows the linear regression corresponding two-sided 95% confidence interval (n = 52). The horizontal grey line denotes zero difference (that is, identical coverage for both methods). Sample sizes: primates $n = 43$, mosquitoes $n = 9$. Pearson's r was calculated using a two-sided test ($r = –0.78$, $p = 1.2 \times 10^{-11}$. We note that the association remained largely unchanged when removing the ID72 (outlier, -64.74% difference; Spearman's r = –0.77, n=51). Given the low Ct (16.82) and strong yield from matched amplicon library, the shortfall during metagenomics is most consistent with a library-specific handling artefact, either barcode under-loading/pool imbalance or inefficient adapter/barcode ligation which would redux demultiplexable viral fragments and explain the very low number of YFV reads.

Supplementary Fig. 4

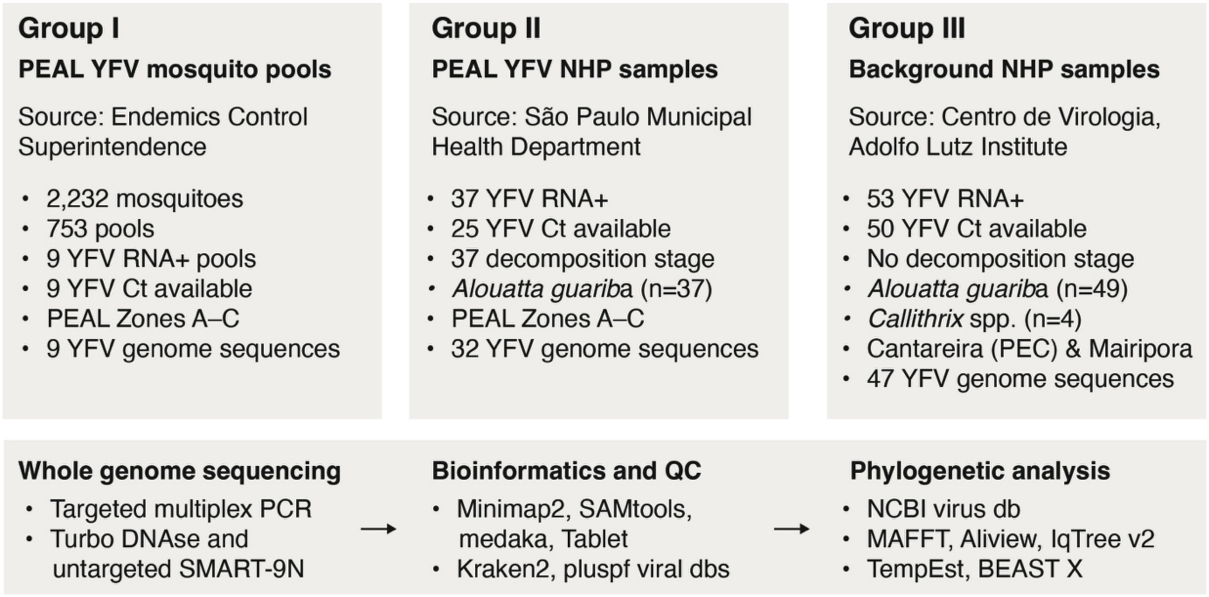

Overview of sample collection, data sources, and sequencing outputs across Groups I, II and III (GI–GIII) samples. NHP=Nonhuman primate. NA=Not available.

Supplementary Fig. 5

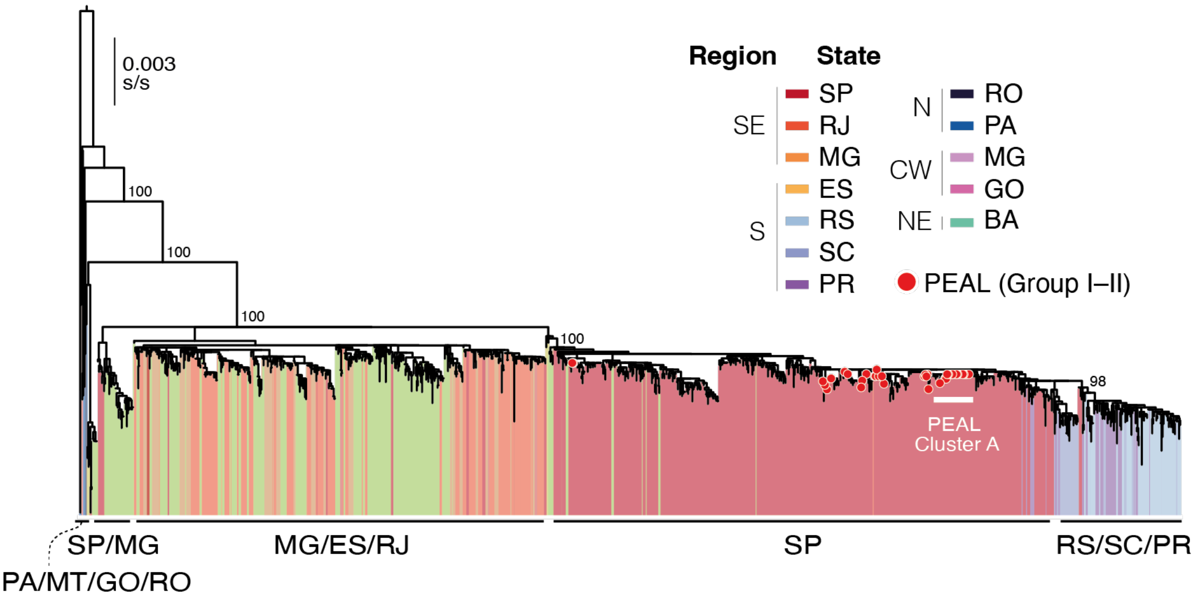

Maximum likelihood phylogeny of YFV genomes from Brazil. The tree was inferred from the YFV "Brazil dataset" (n=1,063). Terminal branches are coloured by the Brazilian state of sampling and grouped by macro-region (SE=Southeast, S=South, CW=Centre-West, N=North, NE=Northeast). State abbreviations are as follows: PA=Pará, MT=Mato Grosso, GO=Goiás, RO=Rondônia, SP=São Paulo, MG=Minas Gerais, ES=Espírito Santo, RJ=Rio de Janeiro, RS=Rio Grande do Sul, SC=Santa Catarina, PR=Paraná. Red circles highlight YFV genomes generated from NHPs and mosquito pools at PEAL (groups I and II). The main lineage containing these sequences is labelled PEAL Cluster A. Numerical values on key internal nodes indicate bootstrap support (%).

Supplementary Fig. 6

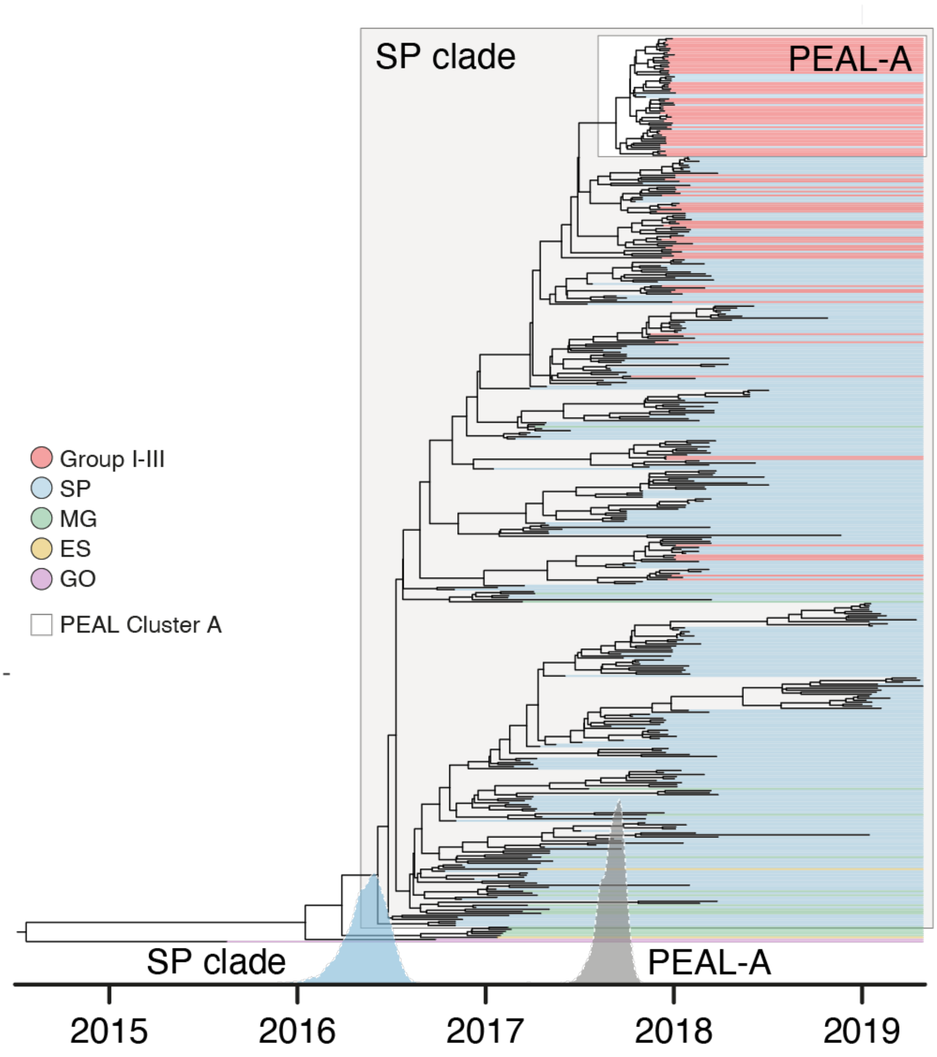

Time-scaled phylogeny of the São Paulo dataset. The Bayesian maximum-clade-credibility tree was reconstructed from 450 YFV genomes sampled predominantly in southeastern Brazil. The x-axis is in calendar years. Terminal branches are coloured according to Brazilian state of sampling (Group I-III sequences are show in red). Grey shaded rectangle delineates the monophyletic YFV_{SP} clade reported previously[1]; white box highlights PEAL Cluster A (PEAL-A) within that clade. KDE curves show the posterior distributions of the TMRCA for the SP clade, and for the PEAL Clade A estimated using this dataset.

Supplementary Fig. 7

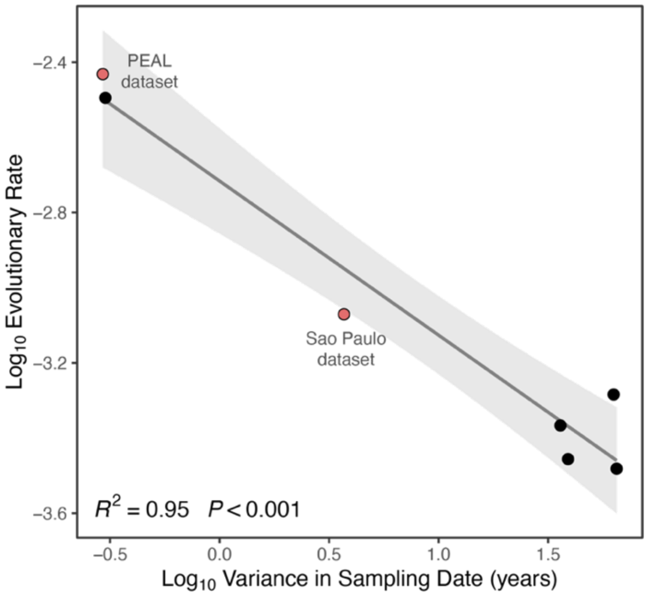

Time-dependency of YFV South American genotype 1 evolutionary rates. Median evolutionary rates obtained for the PEAL and São Paulo datasets are shown in red circles. Estimates from other previously published YFV datasets[2–6] are shown in black circles. Variance in sampling dates ranged between 107 days (PEAL dataset), 3.7 years (São Paulo dataset) to 65 years. Shaded band = 95 % CI.

Supplementary Fig. 8.

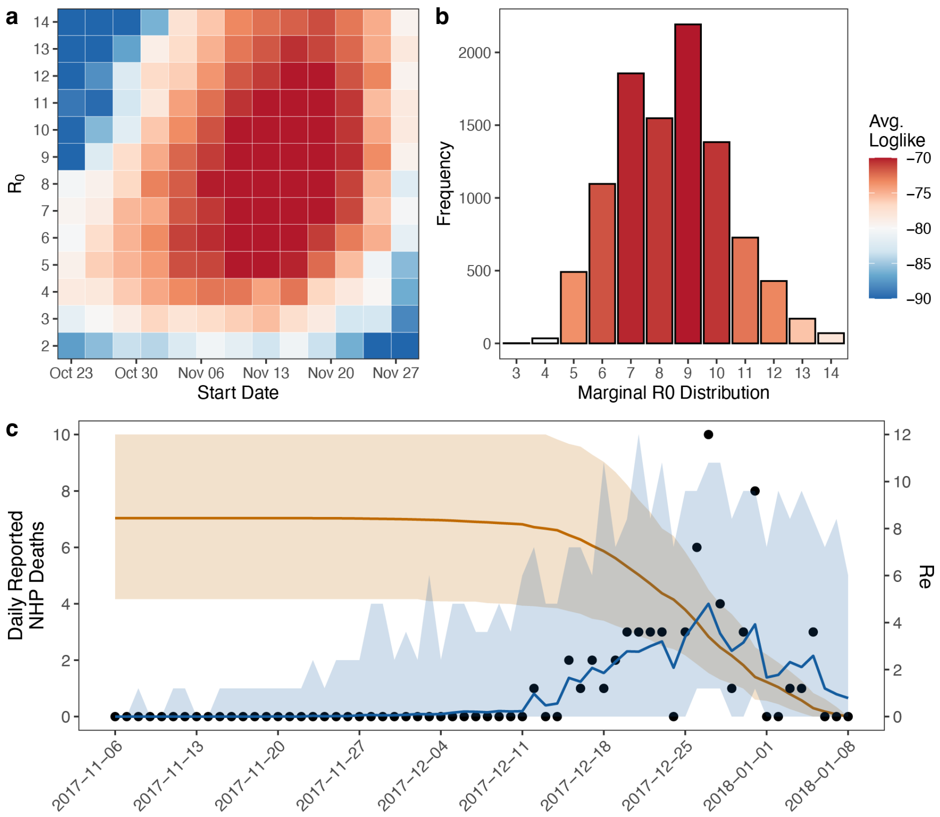

Estimate of the reproduction number and transmission dynamics in PEAL assuming a later timing of importation based on the MRCA of Cluster A. a. Average log-likelihood (colour scale) for each joint combination of the basic reproduction number (R_0, y-axis) and outbreak start date (x-axis). Warmer tones indicate parameter pairs with higher posterior support. **b.** Marginal posterior distribution of R_0 integrating over all start-date hypotheses shown in panel a. Bar height denotes posterior sample frequency; colours match the log-likelihood scale used in panel a. **c.** Model fit to daily reported NHP deaths (black circles, left axis) and the corresponding effective reproduction number over time (R_e, orange line, right axis). Blue line and shaded ribbon give the posterior median and 95% CI for model-predicted deaths; the orange ribbon shows the 95% CI for R_e.

References

1. Hill, S. C. *et al. Climate and Land-Use Shape the Spread of Zoonotic Yellow Fever Virus*. http://medrxiv.org/lookup/doi/10.1101/2022.08.25.22278983 (2022) doi:10.1101/2022.08.25.22278983.
2. Bryant, J. E., Holmes, E. C. & Barrett, A. D. T. Out of Africa: A Molecular Perspective on the Introduction of Yellow Fever Virus into the Americas. *PLoS Pathog* **3**, e75 (2007).
3. Gómez, M. M. *et al.* Genomic and structural features of the yellow fever virus from the 2016–2017 Brazilian outbreak. *Journal of General Virology* **99**, 536–548 (2018).
4. Delatorre, E. *et al.* Distinct YFV Lineages Co-circulated in the Central-Western and Southeastern Brazilian Regions From 2015 to 2018. *Front. Microbiol.* **10**, 1079 (2019).
5. Mir, D. *et al.* Phylodynamics of Yellow Fever Virus in the Americas: new insights into the origin of the 2017 Brazilian outbreak. *Sci Rep* **7**, 7385 (2017).
6. Faria, N. R. *et al.* Genomic and epidemiological monitoring of yellow fever virus transmission potential. *Science* **361**, 894–899 (2018).
