# Supplementary material for: Evolution and spillover dynamics of yellow fever at the forest–urban interface in Brazil
Source: Nat Microbiol. 2026 Mar 11;11(4):877–91. doi: 10.1038/s41564-026-02302-w (PMC13056586; doi:10.1038/s41564-026-02302-w)
Supplement: Supplementary file 2 — Reporting Summary [file 41564_2026_2302_MOESM2_ESM.pdf]

nature portfolio

Corresponding author(s): Juliana Telles de Deus, Luis Filipe Mucci, Nuno R. Faria

Last updated by author(s): Feb 16, 2026

Reporting Summary

Nature Portfolio wishes to improve the reproducibility of the work that we publish. This form provides structure for consistency and transparency in reporting. For further information on Nature Portfolio policies, see our Editorial Policies and the Editorial Policy Checklist.

Statistics

For all statistical analyses, confirm that the following items are present in the figure legend, table legend, main text, or Methods section.

n/a	Confirmed	
☐	☒	The exact sample size (*n*) for each experimental group/condition, given as a discrete number and unit of measurement
☐	☒	A statement on whether measurements were taken from distinct samples or whether the same sample was measured repeatedly
☐	☒	The statistical test(s) used AND whether they are one- or two-sided<br>*Only common tests should be described solely by name; describe more complex techniques in the Methods section.*
☐	☒	A description of all covariates tested
☐	☒	A description of any assumptions or corrections, such as tests of normality and adjustment for multiple comparisons
☐	☒	A full description of the statistical parameters including central tendency (e.g. means) or other basic estimates (e.g. regression coefficient) AND variation (e.g. standard deviation) or associated estimates of uncertainty (e.g. confidence intervals)
☐	☒	For null hypothesis testing, the test statistic (e.g. *F*, *t*, *r*) with confidence intervals, effect sizes, degrees of freedom and *P* value noted<br>*Give P values as exact values whenever suitable.*
☐	☒	For Bayesian analysis, information on the choice of priors and Markov chain Monte Carlo settings
☐	☒	For hierarchical and complex designs, identification of the appropriate level for tests and full reporting of outcomes
☐	☒	Estimates of effect sizes (e.g. Cohen's *d*, Pearson's *r*), indicating how they were calculated

Our web collection on statistics for biologists contains articles on many of the points above.

Software and code

Policy information about availability of computer code

Data collection

Commercial software / platforms:
MinKNOW v1.15.1 (Oxford Nanopore Technologies): control and run execution for GridION sequencing.
Guppy v5.0.16 (Oxford Nanopore Technologies): basecalling, demultiplexing and adapter trimming for Nanopore reads.
ABI 7500 Real⍰Time PCR System (Thermo Fisher Scientific): instrument software for RT⍰qPCR YFV detection (standard ABI7500 environment).

Open⍰source software:
minimap2 v2.28: initial mapping at data⍰collection QC stage (read⍰quality and barcoding checks).
SAMtools v1.20: initial alignment inspection and QC.
NanoStat v1.1.2: sequencing run summaries (read counts, N50)
Tablet (v1.17.08.17): visual inspection of raw read alignments.

Custom and protocol⍰specific code:
SMART⍰9N metagenomics workflow (custom scripts): laboratory protocol–linked scripts for cDNA tagging, sample preparation tracking, barcode assignment.
Custom code related to data collection is deposited in the project’s code repository as listed in the manuscript’s Code Availability section.

Data analysis

Commercial software:
MinKNOW v1.15.1 (ONT): generation of FAST5/FASTQ output; sequencing metadata exported for downstream analysis.
Guppy v5.0.16: high⍰accuracy basecalling and demultiplexing used as inputs for analytical pipelines.

Open⍰source software:

Genome processing and consensus generation
minimap2 v2.28: mapping reads to YFV and HAV references.
SAMtools v1.20: sorting, indexing, depth profiling.
NanoStat v1.1.2: long-read QC summaries.
Tablet (1.17.08.17): manual visual QC.
medaka v1.12.1: variant calling.

Taxonomic and host/vector identification:
BLAST+ (2.16.0): host/vector confirmation by BLASTn.
Kraken2 v2.1.3 with RefSeq viral genomes database: pathogen-agnostic classification.
pavian: classification visualisation.

Phylogenetics and evolutionary analysis:
MAFFT v7 (--auto): multiple sequence alignment.
IQ-TREE2 v2.3.6 with ModelFinder Plus, UFBoot2 and SH-aLRT: maximum-likelihood phylogenetics.
TempEst v1.5.3: temporal signal assessment.
BEAST X (BEAGLE v3 acceleration): Bayesian time-scaled phylogenetics; XML configurations included in repository.

Statistical modelling and visualisation (R ecosystem):
R v4.3.2 for all modelling.
tidyverse: wrangling and plotting.
lubridate: temporal handling.
MASS (glm.nb): negative-binomial GLMs.
corrplot v0.95: correlation diagnostics.
stargazer: regression table formatting.
ggplot2: statistical visualisation.
broom v1.0.10: tidy model outputs.

Transmission modelling and inference:
Stan (v2.36.0): Bayesian fitting of incubation-period and delay distributions.
individual R package (v1.1.17): simulation framework for the individual-based model.
Custom R and bash scripts for particle filtering, likelihood estimation, branching-process simulations, model visualisation and reproduction-number estimation, available under Code Availability.

Custom code:
Custom code for the IBM transmission model, particle-filtering code, R analysis pipelines, phylogenetic XMLs and plotting scripts is deposited in a dedicated repository as specified in the Code Availability section.

For manuscripts utilizing custom algorithms or software that are central to the research but not yet described in published literature, software must be made available to editors and reviewers. We strongly encourage code deposition in a community repository (e.g. GitHub). See the Nature Portfolio guidelines for submitting code & software for further information.

Data

Policy information about availability of data

All manuscripts must include a data availability statement. This statement should provide the following information, where applicable:

- Accession codes, unique identifiers, or web links for publicly available datasets
- A description of any restrictions on data availability
- For clinical datasets or third party data, please ensure that the statement adheres to our policy

The YFV genomic data generated in this study are available in NCBI GenBank under accession numbers OQ714241–OQ714328. The near-complete hepatitis A virus (HAV) genome is available in NCBI GenBank under accession number PV702359. Raw metagenomic sequencing reads (FASTQ files) have been deposited in the NCBI Sequence Read Archive under accession numbers SAMN48792130–SAMN48792218 (BioProject PRJNA1269522). Detailed laboratory protocols, including the viral metagenomic sequencing workflow and SMART-9N primer sequences, are available at https://protocols.io46. The phylogenetic trees, genomic datasets, and XML files are publicly accessible in our dedicated GitHub repository (https://github.com/cadde-centre/YFV_horto).

Research involving human participants, their data, or biological material

Policy information about studies with human participants or human data. See also policy information about sex, gender (identity/presentation), and sexual orientation and race, ethnicity and racism.

Reporting on sex and gender	N/A.
Reporting on race, ethnicity, or other socially relevant groupings	N/A.
Population characteristics	N/A.
Recruitment	N/A.
Ethics oversight	N/A.

Note that full information on the approval of the study protocol must also be provided in the manuscript.

Field-specific reporting

Please select the one below that is the best fit for your research. If you are not sure, read the appropriate sections before making your selection.

☐ Life sciences ☐ Behavioural & social sciences ☒ Ecological, evolutionary & environmental sciences

For a reference copy of the document with all sections, see nature.com/documents/nr-reporting-summary-flat.pdf

Ecological, evolutionary & environmental sciences study design

All studies must disclose on these points even when the disclosure is negative.

Study description	We investigated a sylvatic yellow fever outbreak in a forest fragment in metropolitan São Paulo by integrating mosquito sampling, detection of naturally deceased howler monkeys, and metagenomic sequencing with phylogenetic and epidemiological analyses.
Research sample	Naturally deceased Alouatta guariba found in PEAL, plus a small number of background NHP cases from Cantareira (routine state surveillance). Free-flying forest mosquitoes, mainly Haemagogus leucocelaenus, were also collected across PEAL.
Sampling strategy	All detected NHP carcasses were sampled. Mosquitoes were collected at fixed sites using hand nets, aspirators and CO_2 traps at ground and canopy levels. Sample sizes reflect natural encounter rates; no sample?size calculations were applicable.
Data collection	Field teams georeferenced NHP carcasses and collected mosquitoes using standard entomological methods. Samples were preserved and processed in accredited public-health laboratories following standard protocols for RNA extraction and metagenomic sequencing.
Timing and spatial scale	Sampling occurred between Oct 2017 and Oct 2018 in PEAL, with 43 mosquito-sampling days and continuous NHP monitoring. Additional contextual NHP sequences originated from neighbouring Cantareira State Park.
Data exclusions	Only samples that failed objective quality criteria (e.g., insufficient tissue, <70% genome coverage for phylogenetics) were excluded. Exclusion criteria were pre-defined.
Reproducibility	Field and laboratory methods followed standardised protocols. All analytical code, parameters and workflows are version-controlled and publicly available for full reproducibility.
Randomization	Not applicable. Samples reflect natural mortality and natural mosquito abundance; no individuals were allocated to treatment groups.
Blinding	Not applicable. All samples were processed using identical laboratory and analytical workflows regardless of origin or expected YFV status.

Did the study involve field work? ☒ Yes ☐ No

Field work, collection and transport

Field conditions	Field work was conducted in Parque Estadual Alberto Löfgren (PEAL), a 186-ha Atlantic Forest fragment embedded within metropolitan São Paulo, Brazil. Sampling took place between October 2017 and October 2018 under warm, humid subtropical conditions. Mean daily temperatures ranged from 18–28 °C, with seasonal variation described using ERA5-Land data. Mosquito surveys were performed between 09:00 and 15:00 across forest interior, forest–urban edges, and canopy strata. Terrain included dense forest cover, steep gradients, and mixed vegetation typical of the Cantareira mountain foothills.
Location	Sampling occurred mostly within PEAL State Park (23.466–23.455° S, 46.655–46.630° W; 775–850 m elevation), in the northern zone of São Paulo city. Field sites covered management Zones A–C, including forest interior, the Vila Amália settlement edge, peridomestic surroundings, and trails near the Serra da Cantareira. Non-human primate carcasses and mosquito collections were georeferenced and spatially restricted to areas managed by the São Paulo State Secretariat for the Environment. In addition to PEAL field samples, a small number of non-human primate background cases from the contiguous Cantareira State Park (PEC), collected independently under routine state surveillance, were included for phylogenetic context. No additional fieldwork was carried out by the authors in PEC.
Access & import/export	Field activities were carried out exclusively within Parque Estadual Alberto Löfgren (PEAL) under authorisation from the São Paulo State Secretariat for the Environment and in accordance with Brazilian Ministry of Health guidelines for wildlife surveillance. Sampling of non-human primate carcasses followed approved protocols from the Instituto Adolfo Lutz Ethics Committee for Animal Use in Research (0135D/2012; 020G/2014). Additional non-human primate background samples from the contiguous Cantareira State Park (PEC) were not collected by the authors, but obtained from routine state surveillance systems managed by the São Paulo State environmental and health authorities. These samples were provided directly to the reference laboratories responsible for YFV monitoring. All specimens remained in Brazil and were processed in São Paulo, Brazil (Instituto Adolfo Lutz and the University of São Paulo). No international transport or export permits were required.

Disturbance	Disturbance to wildlife and habitat was minimised by restricting all field sampling to naturally deceased non?human primates and by using low?impact mosquito?collection methods (hand nets, aspirators, CO_2baited traps) along predefined trails and designated access points. No animals were captured, handled, immobilised or euthanised for this study. Work was conducted by trained personnel who avoided sensitive habitat, minimised noise and foot traffic, and adhered to conservation and biosafety guidelines for protected Atlantic Forest areas. Background PEC samples used for phylogenetic context were derived from routine state surveillance, and no field activities were conducted by the authors in PEC, ensuring no additional disturbance in that conservation unit.

Reporting for specific materials, systems and methods

We require information from authors about some types of materials, experimental systems and methods used in many studies. Here, indicate whether each material, system or method listed is relevant to your study. If you are not sure if a list item applies to your research, read the appropriate section before selecting a response.

Materials & experimental systems

n/a	Involved in the study	
☐	☐	Antibodies
☐	☐	Eukaryotic cell lines
☐	☐	Palaeontology and archaeology
☐	☒	Animals and other organisms
☐	☐	Clinical data
☐	☐	Dual use research of concern
☐	☐	Plants

Methods

n/a	Involved in the study	
☐	☐	ChIP-seq
☐	☐	Flow cytometry
☐	☐	MRI-based neuroimaging

Antibodies

Antibodies used	N/A.
Validation	N/A.

Eukaryotic cell lines

Policy information about cell lines and Sex and Gender in Research

Cell line source(s)	N/A.
Authentication	N/A.
Mycoplasma contamination	N/A.
Commonly misidentified lines (See ICLAC register)	N/A.

Palaeontology and Archaeology

Specimen provenance	N/A.
Specimen deposition	N/A.
Dating methods	N/A.

☐ Tick this box to confirm that the raw and calibrated dates are available in the paper or in Supplementary Information.

Ethics oversight	N/A.

Note that full information on the approval of the study protocol must also be provided in the manuscript.

Animals and other research organisms

Policy information about studies involving animals; ARRIVE guidelines recommended for reporting animal research, and Sex and Gender in Research

Laboratory animals	N/A. No laboratory animals were used.

Wild animals	Only naturally deceased NHP carcasses found through routine state surveillance were sampled. No animals were captured, handled or euthanised.
Reporting on sex	Sex was not consistently identifiable for many carcasses due to decomposition, and sex was not part of the study design. Analyses therefore were not sex?stratified.
Field-collected samples	Only naturally deceased NHP carcasses were sampled for YFV surveillance.
Ethics oversight	The surveillance protocol for dead NHPs was approved by the Ethics Committee for the use of Animals in Research, Instituto Adolfo Lutz, under the numbers 0135D/2012 and 020G/2014, which include work in protected environmental areas.

Note that full information on the approval of the study protocol must also be provided in the manuscript.

Clinical data

Policy information about clinical studies

All manuscripts should comply with the ICMJE guidelines for publication of clinical research and a completed CONSORT checklist must be included with all submissions.

Clinical trial registration	N/A.
Study protocol	N/A.
Data collection	N/A.
Outcomes	N/A.

Dual use research of concern

Policy information about dual use research of concern

Hazards

Could the accidental, deliberate or reckless misuse of agents or technologies generated in the work, or the application of information presented in the manuscript, pose a threat to:

No	Yes	
☒	☐	Public health
☒	☐	National security
☒	☐	Crops and/or livestock
☒	☐	Ecosystems
☒	☐	Any other significant area

Experiments of concern

Does the work involve any of these experiments of concern:

No	Yes	
☒	☐	Demonstrate how to render a vaccine ineffective
☒	☐	Confer resistance to therapeutically useful antibiotics or antiviral agents
☒	☐	Enhance the virulence of a pathogen or render a nonpathogen virulent
☒	☐	Increase transmissibility of a pathogen
☒	☐	Alter the host range of a pathogen
☒	☐	Enable evasion of diagnostic/detection modalities
☒	☐	Enable the weaponization of a biological agent or toxin
☒	☐	Any other potentially harmful combination of experiments and agents

Plants

Seed stocks	N/A.
Novel plant genotypes	N/A.
Authentication	N/A.

ChIP-seq

Data deposition

- [ ] Confirm that both raw and final processed data have been deposited in a public database such as GEO.
- [ ] Confirm that you have deposited or provided access to graph files (e.g. BED files) for the called peaks.

Data access links *May remain private before publication.*	N/A.
Files in database submission	N/A.
Genome browser session (e.g. UCSC)	N/A.

Methodology

Replicates	N/A.
Sequencing depth	N/A.
Antibodies	N/A.
Peak calling parameters	N/A.
Data quality	N/A.
Software	N/A.

Flow Cytometry

Plots

Confirm that:

- [ ] The axis labels state the marker and fluorochrome used (e.g. CD4-FITC).
- [ ] The axis scales are clearly visible. Include numbers along axes only for bottom left plot of group (a 'group' is an analysis of identical markers).
- [ ] All plots are contour plots with outliers or pseudocolor plots.
- [ ] A numerical value for number of cells or percentage (with statistics) is provided.

Methodology

Sample preparation	N/A.
Instrument	N/A.
Software	N/A.
Cell population abundance	N/A.

Gating strategy | N/A.

☐ Tick this box to confirm that a figure exemplifying the gating strategy is provided in the Supplementary Information.

Magnetic resonance imaging

Experimental design

Design type: N/A.

Design specifications: N/A.

Behavioral performance measures: N/A.

Acquisition

Imaging type(s): N/A.

Field strength: N/A.

Sequence & imaging parameters: N/A.

Area of acquisition: N/A.

Diffusion MRI ☐ Used ☐ Not used

Preprocessing

Preprocessing software: N/A.

Normalization: N/A.

Normalization template: N/A.

Noise and artifact removal: N/A.

Volume censoring: N/A.

Statistical modeling & inference

Model type and settings: *Specify type (mass univariate, multivariate, RSA, predictive, etc.) and describe essential details of the model at the first and second levels (e.g. fixed, random or mixed effects; drift or auto-correlation).*

Effect(s) tested: *Define precise effect in terms of the task or stimulus conditions instead of psychological concepts and indicate whether ANOVA or factorial designs were used.*

Specify type of analysis: ☐ Whole brain ☐ ROI-based ☐ Both

Statistic type for inference (See Eklund et al. 2016): *Specify voxel-wise or cluster-wise and report all relevant parameters for cluster-wise methods.*

Correction: *Describe the type of correction and how it is obtained for multiple comparisons (e.g. FWE, FDR, permutation or Monte Carlo).*

Models & analysis

n/a	Involved in the study
☐	☐ Functional and/or effective connectivity
☐	☐ Graph analysis
☐	☐ Multivariate modeling or predictive analysis

Functional and/or effective connectivity: *Report the measures of dependence used and the model details (e.g. Pearson correlation, partial correlation, mutual information).*

Graph analysis: *Report the dependent variable and connectivity measure, specifying weighted graph or binarized graph, subject- or group-level, and the global and/or node summaries used (e.g. clustering coefficient, efficiency, etc.).*

Multivariate modeling and predictive analysis

Specify independent variables, features extraction and dimension reduction, model, training and evaluation metrics.
